# Automated Stitching of Microtubule Centerlines across Serial Electron Tomograms

**DOI:** 10.1371/journal.pone.0113222

**Published:** 2014-12-01

**Authors:** Britta Weber, Erin M. Tranfield, Johanna L. Höög, Daniel Baum, Claude Antony, Tony Hyman, Jean-Marc Verbavatz, Steffen Prohaska

**Affiliations:** 1 Zuse Institute Berlin, Berlin, Germany; 2 European Molecular Biology Laboratory, Heidelberg, Germany; 3 Max Planck Institute for Molecular Biology and Genetics, Dresden, Germany; 4 Sir William Dunn School of Pathology, University of Oxford, Oxford, United Kingdom; Stanford University School of Medicine, United States of America

## Abstract

Tracing microtubule centerlines in serial section electron tomography requires microtubules to be stitched across sections, that is lines from different sections need to be aligned, endpoints need to be matched at section boundaries to establish a correspondence between neighboring sections, and corresponding lines need to be connected across multiple sections. We present computational methods for these tasks: 1) An initial alignment is computed using a distance compatibility graph. 2) A fine alignment is then computed with a probabilistic variant of the iterative closest points algorithm, which we extended to handle the orientation of lines by introducing a periodic random variable to the probabilistic formulation. 3) Endpoint correspondence is established by formulating a matching problem in terms of a Markov random field and computing the best matching with belief propagation. Belief propagation is not generally guaranteed to converge to a minimum. We show how convergence can be achieved, nonetheless, with minimal manual input. In addition to stitching microtubule centerlines, the correspondence is also applied to transform and merge the electron tomograms. We applied the proposed methods to samples from the mitotic spindle in *C. elegans*, the meiotic spindle in *X. laevis*, and sub-pellicular microtubule arrays in *T. brucei*. The methods were able to stitch microtubules across section boundaries in good agreement with experts' opinions for the spindle samples. Results, however, were not satisfactory for the microtubule arrays. For certain experiments, such as an analysis of the spindle, the proposed methods can replace manual expert tracing and thus enable the analysis of microtubules over long distances with reasonable manual effort.

## Introduction

**Figure 1:**
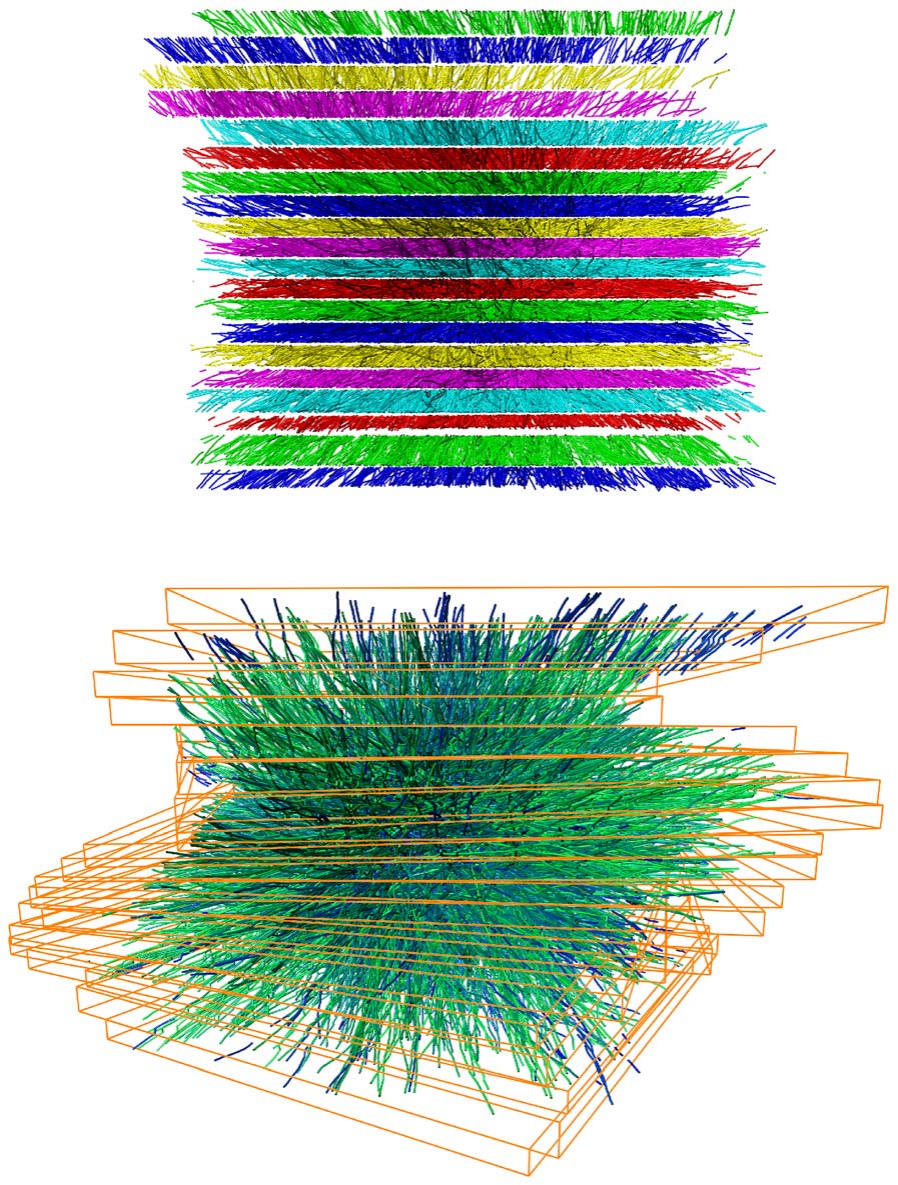
Starting point and output of our computational methods. Results are displayed for a stack of 3 00 nm thick and 5
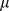
m wide sections of microtubule centerlines in the mitotic spindle of a *C. elegans* early embryo. Each section contains approximately 1500 microtubules, which were traced in electron tomograms. Top: Side view of unaligned centerlines before stitching. Colors indicate different sections. Bottom: Perspective view of the stitched microtubules after applying our algorithms. Color indicates length of the stitched microtubules: greenish lines are longer; bluish lines are shorter. The orange bounding boxes help getting an impression of the alignment transformation. The boxes were oriented parallel to the main coordinate axes before alignment. Some lines do not have a continuation in the next section (for example bluish lines at top right), because the area covered by the tomograms varied from section to section.

The problem of stitching lines arises when filamentous or tubular structures, such as microtubules, need to be traced across multiple consecutive semi-thin (a few hundred nanometer thick) sections, each of which contains only a portion of the filaments. Three-dimensional images of such sections can be obtained, for example, by electron tomography. Due to sample processing, however, the sections are often individually rotated, shifted, scaled, or deformed in a non-linear way. In this paper, we focus on the stitching of microtubules segmented from serial section electron tomograms. Within each section, microtubules are represented as polygonal centerlines. For microtubules that traverse the entire thickness of the section, the lines have two endpoints at or close to the two section boundaries. Endpoints may also occur further away from the section boundary for microtubules that naturally end there. Stitching consists of aligning sections, matching corresponding endpoints at two facing section boundaries, and connecting corresponding lines across multiple sections. See Figure 1 for an example that illustrates the complexity of the task and a solution achieved with our methods. Manual stitching of microtubule centerlines is labor intensive, and sometimes it is even impossible for a human to reliably decide how to align sections and how to connect the lines. Automated processing would be an attractive alternative, in particular, because the volumes reconstructed using electron tomography have been constantly increasing towards covering full cells (for example, Höög et al. [2], [3]), or large dense structures like centrosomes during cell division (for example, Müller-Reichert et al. [4], O'Toole et al. [5]). A purely manual segmentation of even larger structures, such as the massive meiotic spindle of *X. laevis* (see Brugués et al. [6], Loughlin et al. [7]), seems unrealistic.

Automatic stitching of microtubule endpoints from consecutive sections is challenging. The samples suffer from shearing and material loss at the boundary of sections during cutting and image acquisition. Samples shrink, warp and twist as a consequence of the electron exposure (see Figure S4 and also Luther [8]). Furthermore, automated and manual segmentation, too, will not always perfectly capture the endpoints (see Weber et al. [1]). Since microtubules, on the other hand, can be as close to each other as 25 nm (Lacomble et al. [9], Höög et al. [2], [10], McDonald et al. [11], Ding et al. [12]), the correspondence of endpoints of close-by microtubules may be difficult to decide. A stitching method must handle the described deformations and be robust in the presence of noise.

To the best of our knowledge, the only software for stitching microtubule endpoints using the segmented centerlines is a semi-automatic tool integrated into the IMOD software (Kremer and Mastronarde [13]). Tomograms are roughly aligned using manually selected landmarks that are used to correct the deformation with a thin-plate spline model. Microtubules are traced afterwards in the aligned tomograms. Identified microtubules are then used to refine the deformation correction. This approach has been used successfully, for example, by McIntosh et al. [14], O'Toole et al. [5], [15], and Höög et al. [16]. However, selecting matching microtubules manually is time consuming, tedious and, depending on the contrast of the tomogram and the presence of features to guide the user to potential matches, it can be infeasible.

An automated method could facilitate the stitching of microtubule centerlines over long distances in a reliable manner. Assuming that microtubule centerlines have been traced for a stack of electron tomograms of consecutive serial sections, two related problems need to be solved: 1) The sections need to be aligned, that is they need to be rotated and translated, and local distortions that are caused by tomogram acquisition need to be corrected. 2) Corresponding microtubules need to be matched and connected across section boundaries. If all centerlines and a perfect alignment were available, the matching should be obvious, because corresponding endpoints should be closer to each other than to any other endpoint. If a perfect endpoint matching was known, it could be used to compute an alignment such that the matched endpoints would be transformed onto each other. In practice, however, a perfect solution cannot be expected due to the limited data quality.

Solutions to the alignment and the matching problem have been studied before. A common method for finding an initial alignment of points is to identify a subset of matching points using transformation invariant features and then compute the initial alignment from these pairs. Point pairs can be sampled, for example, with RANSAC (see Fischler and Bolles [17]) based on a local feature descriptor such as the sorted distance to neighboring points (see for example Lee et al. [18]). This method has been successfully applied in point registration in biology (Preibisch et al. [19]), image registration (Saalfeld et al. [20]), and to register lines (Yao et al. [21]). RANSAC-based methods have been demonstrated to perform well even in the presence of noise, outliers and strong deformation.

If only a few points need to be aligned, the problem can be formulated as finding cliques in a graph representation, called the distance compatibility graph (DCG). This method has been used for molecular shape analysis (for example Baum et al. [22]). It has also been applied for matching of neuron ends (for example Dercksen et al. [23]).

The most popular algorithm for the registration of points that are already coarsely aligned is the iterative closest points algorithm (reviewed in Rusinkiewicz and Levoy [24]). A probabilistic variant that formulates the problem in terms of a Gaussian mixture model was proposed by several authors (Rangarajan et al. [25], Wells [26]). The probabilistic variant has many advantages, including that the deviation of the assumed Gaussian mixture model can serve as a measure of uncertainty of the result; that the method naturally deals with outliers; and that the method is easy to implement. The approach has also been adapted for solving elastic deformation models (Jian and Vermuri [27], Myronenko and Song [28]).

Once the points are aligned, a common approach to finding unambiguously corresponding pairs is to compute a maximum weighted matching (MWM) on a bipartite graph (see Kuhn [29]). If a bipartite graph cannot capture enough prior information about the data, the matching can also be formulated in terms of a Markov random field (see Koller and Friedman [30]). This has received a lot of attention (for example, Caetano et al. [31], Sanghavi et al. [32]) and has been successfully applied to many applications, the closest to ours being the one described by Amat et al. [33], where the point matching computation is used to find a proper alignment of series of transmission electron microscopy images using the gold particles on top of the sample for alignment.

**Figure 2:**
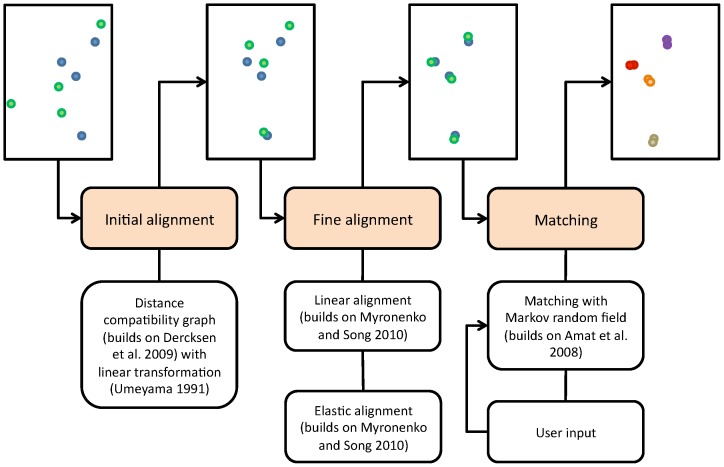
Computational methods. Top and middle: Illustration of two neighboring sections (view from top) and main processing steps. Bottom: algorithmic details and references. Endpoints are illustrated in blue and green. The initial alignment computes a coarse, linear alignment from a subset of endpoints. The fine alignment moves endpoints closer in two steps: a linear alignment followed by an elastic alignment. The matching determines pairs of corresponding endpoints (indicated in different colors), which are finally connected across sections (not illustrated).

In the following, we present computational methods for the alignment and matching of microtubule centerlines that build upon the described work. See Figure 2 for an overview. 1) For the initial coarse alignment, we identify cliques in a distance compatibility graph. 2) For a fine alignment of the endpoints, we extend Myronenko and Song's work [28] by integrating line orientation. 3) For establishing correspondences between endpoints, we formulate a matching problem in terms of a Markov random field similar to Amat et al.'s [33]. Matchings are computed with belief propagation on a factor graph. Because belief propagation does not necessarily converge to a minimum in general, we propose a scheme to seek user input to resolve non-converging cases. With little, although carefully selected input, convergence is achieved for our application. We demonstrate the utility of the approach on electron tomograms of microtubules in *Caenorhabditis elegans* mitotic spindles in early embryos (*C. elegans*), *Xenopus laevis* oocyte meiotic spindles (*X. laevis*) and the sub-pellicular microtubule skeleton of *Trypanosoma brucei* (*T. brucei*). The combination of sample preparation and computational methods allows automated stitching of microtubule centerlines with less that 5% connections that disagree with an expert's opinion at each section boundary.

## Materials and Methods

### Sample preparation

Three different types of samples were used. Samples were prepared using well-established protocols. 1) Bipolar spindles were assembled from *X. laevis* egg extract using the previously published protocols Hannak and Heald [34] and Murray [35]. In brief, eggs collected from adult frogs were cleaned, de-jellied and centrifuged at 16,488 rcf for 12 min. 100 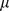
l of the collected cytosolic fraction, which is naturally arrested in metaphase, was sent to interphase with the addition of 0.4mM calcium and sperm nuclei. Once in interphase, equal volumes of the collected cytosolic fraction and the now interphasic fraction were mixed with 1 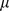
l of Cy3-labelled tubulin to generate bipolar spindles. Animal use was approved by The Institutional Animal Care and Use Committee of The European Molecular Biology Laboratory (Permit Number: CA0555005). 2) As described in Müller-Reichert et al. [36], *C. elegans* (Bristol N2) expressing GFP-tagged tubulin were cut in half and individual single-cell embryos were extracted and sucked into capillary tubes (Leica) cut to 1mm length. The capillary tubes were placed in 100-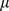
m-deep membrane carriers (Leica) in M9 buffer containing 20% BSA. The cell cycle of embryos was followed by fluorescence miscroscopy until metaphase was reached. 3) As decribed in Höög et al. [37], *T. brucei* cells were grown at logarithmic phase in HMI-9 medium supplemented with 15% (v/v) HIFCS at 37°C. Cells were pelleted by gentle centrifugation (600g). A few microliters of pellet were transferred to membrane carriers.

The specimens (*X. laevis*, *C. elegans* embryos or *T. brucei* cells) were high-pressure frozen using a Leica EMPACT2 or HPM-010 high-pressure freezer, and freeze-substituted in a Leica AFS2. The substitution cocktail contained 2% uranyl acetate in acetone for *T. brucei*; 1% osmium tetroxide, 0.1% uranyl acetate in acetone for *C. elegans*; and 0.1% Tannic Acid, 0.2% glutaraldehyde, 2.5% water in acetone followed by 1% osmium tetroxide, 0.1% uranyl acetate in acetone for *X. laevis*. Specimens were thawed, infiltrated and embedded in epoxy (Epon) or metacrylate (HM20) resins. Semi-thin (300–350 nm thickness) serial sections were cut and collected on formvar-coated slot copper grids for EM. *T. brucei* samples were post-stained with 2% aqueous uranyl acetate (8 min) followed by Reynold's lead citrate [38] (3 min). *C. elegans* samples were post-stained in 2% uranyl acetate in 70% methanol (15 min) followed by lead citrate (5 min). *X. laevis* sections were post-stained only with lead citrate (12 min).

### Electron tomography

10 or 15 nm colloidal gold fiducial particles were deposited on the grids before imaging. Electron tomography tilt series were acquired in a Tecnai F30 electron microscope (FEI Company Ltd., Eindhoven, The Netherlands) operated at 300 kV at 1° tilt increments between 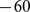 and 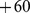 degrees, with the SerialEM software (Mastronarde [39]) using a Gatan US1000 2k camera or a FEI 4k Eagle camera. The pixel size was 1–2.5 nm. To minimize sample shrinkage during acquisition, the samples were pre-irradiated with a dose of 2000 electrons per square Angstrom or more, resulting in pre-shrinkage of the sample. During tomogram acquisition, the samples were submitted to a smaller or equivalent dose of electrons as during pre-irradiation. The tomograms were reconstructed and flattened using the IMOD software package as described in Kremer et al. [13]. Standard settings for plastic tomography were used for the fine alignment of 2D projections, including corrections for rotation, magnification changes due to defocus, distortions, and tilt angles, based on the tracking of gold fiducials on two surfaces of the grids. Due to the large area of interest, 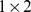 to 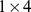 frame montages were acquired for *T. brucei*; for *X. laevis*, 3×3 montages (11.4 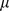
m
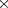
11.4 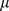
m) were acquired 3 or 4 times per section, before being joined into large supermontages (11.4 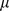
m
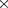
32 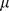
m).

### Microtubule tracing

Microtubule centerlines were traced for each section individually from tomograms that had not been aligned between sections. For the *X. laevis* and *C. elegans* samples, centerlines were traced automatically as described by Weber et al. [1]. The supermontage data was binned prior to tracing. Automatically traced segments were manually validated and corrected using Amira [40]. The segmentation for *T. brucei* was performed manually by an expert using the IMOD software (Kremer et al. [13]).

### Computational methods overview

The input to the computational methods are centerlines from unaligned sections. The final result is a pairwise alignment of sections and a matching of endpoints that is applied to connect corresponding lines across sections.

**Figure 3:**
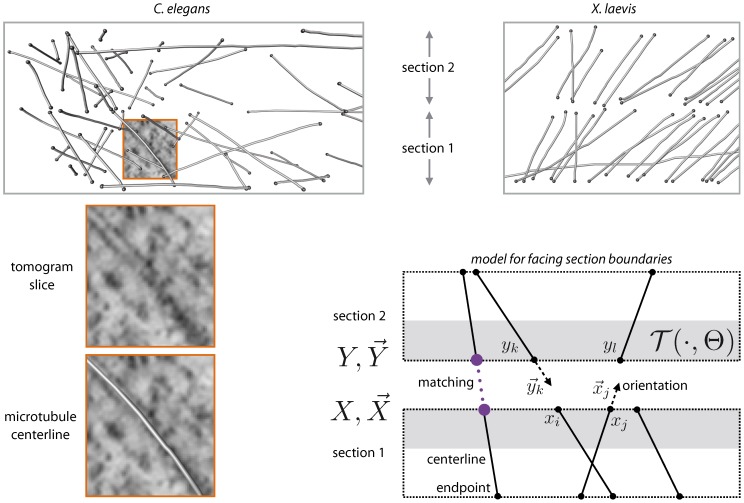
Model for section boundary. Top: View from side at traced microtubule centerlines for two small regions from two neighboring sections of a *C. elegans* sample and a *X. laevis* sample. For the purpose of illustration, the sections have been aligned. Many microtubules are regularly oriented in parallel bundles in the *X. laevis* sample. Microtubules are less regularly oriented in the *C. elegans* sample. Bottom left: Part of a centerline together with the corresponding tomogram slice. Bottom right: Model for boundary between two neighboring sections. Microtubule centerlines are modeled as straight lines with a single orientation (illustrated as dashed arrows). Endpoints close to a boundary (gray area) are described by two-dimensional coordinates as if they were located in a single plane. The transformation 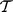 acts on one section boundary (the 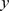
's in section 2). A matching consists of pairs of corresponding endpoints (one pair indicated as dotted line). The goal is to find a transformation and a matching such that corresponding centerlines can be connected across several sections. See main text for detailed notation.

The algorithms use a model for two facing section boundaries as illustrated in Figure 3. Section boundaries are modeled as parallel z-planes. The underlying assumptions are that sections can be cut reasonably parallel and that deformations during tomogram acquisition were minimized by a) coating the grids with carbon to minimize beam damage and sample distortion, b) pre-irradiating a wide area of the sample before tomogram acquisition to minimize local deformations during acquisition, c) by flattening the tomograms after reconstruction. Every section tomogram was visually inspected for flatness and corrected in IMOD or Amira if necessary. Top and bottom tomogram boundaries, therefore, were nearly parallel z-planes before microtubule tracing.

We assume that microtubules cross the section boundary as straight lines. This seems reasonable for microtubules that are perpendicular to the section boundaries, since such microtubules do not curve much over the thickness of a section (300–350 nm). The straightness assumption may be problematic, however, for microtubules that are parallel or nearly parallel to the section boundaries. In practice, the model should be reasonable if enough microtubules are sufficiently perpendicular. Results should be carefully evaluated, though, for regions were microtubules seem to be oriented mostly parallel to the section boundaries.

The endpoints are modeled as points that are located exactly at the section boundary. For microtubules that cross the section boundary, it seems reasonable to assume that they end exactly at the boundary even though real centerlines may contain some positional errors. Microtubules, however, may also naturally end within a section. In practice, the model should be reasonable if more microtubules cross the section boundary than end within a section. The algorithms include means to handle outliers. We simply treat all endpoints as if they ended at the boundary and leave it to the algorithms to handle endpoints without corresponding endpoint in the next section. *X. laevis* samples seem to meet the assumption well (the centerlines displayed in Figure 3 are a typical example). But also for the *C. elegans* samples, which contain many short lines close to the centrosome center, we have not observed problems in practice; the assumption seems to be sufficiently fulfilled.

The final result is computed in three main steps as illustrated in Figure 2. During initial alignment, each section pair is coarsely aligned using a linear transformation. During fine alignment, first a refined linear transformation is applied to each section pair followed by an elastic transformation to correct deformations. The matching identifies corresponding endpoints across the section boundaries, which is finally used to connect lines across multiple sections. We apply all steps in order they are described unless explicitly stated otherwise.

#### Notation

Since we assume that all microtubule endpoints lie in a plane, their positions can be described by two-dimensional coordinates. The 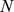 endpoint positions 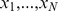 with 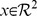 are collectively denoted by 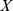 and the 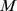 endpoint positions 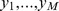 in the neighboring section by 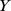. The corresponding line orientations 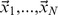 with 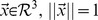 are denoted by 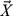 and the orientations of the neighboring section 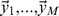 by 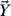. 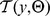 denotes a transformation with parameters 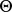. For example, if 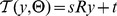 with scaling 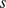, a rotation matrix 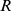 and a translation 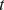, then 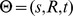. For the fine alignment, we will introduce a continuous random variable 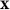 and a discrete random variable 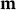 that has states 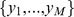. We will write 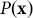 to refer to the probability density function of 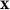 and abbreviate the probability of 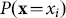 by 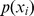. Similarly we will write 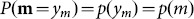 for the discrete random variable. When we describe the Markov random field for endpoint matching, we will introduce one discrete random variable 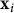 for each endpoint.

### Initial alignment

The *initial alignment algorithm* computes a linear transformation of the two sets of endpoints 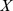 and 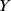 by finding cliques in a distance compatibility graph (DCG) (see Dercksen et al. [23]). Each clique of the DCG establishes a one-to-one correspondence between some points from 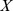 and 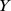, which can be used to compute an alignment. The method works by identifying similar spatial patterns in 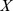 and 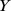. The nodes of the DCG are pairs 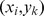 of endpoints from 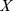 and 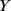. Two DCG nodes 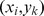 and 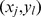 are connected by an edge if 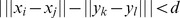, where 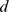 is a threshold 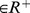. Such a graph is called a 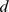-bounded DCG. In the worst case, the memory requirement for building the graph is 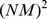, since there are 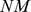 nodes and each of these nodes can be connected to every other node. In practical applications, however, it is much smaller. Now consider a maximal connected subgraph (clique) in the DCG. Because all nodes in this subgraph are mutually connected, all distances 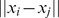 of endpoints from 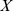 in the clique are close to the respective distances 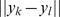 of the endpoints from 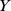. Therefore the endpoints 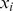 in the clique and the paired 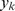 are arranged in a similar spatial pattern. To find a good initial alignment, we search for large cliques in the DCG. This method is only successful if the distortions of the point sets are small.

For the alignment of microtubule endpoints, we can further restrict the number of edges in the DCG by taking into account the line orientations. Thus, we only connect two nodes by an edge in the DCG if 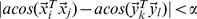. With this modification, we find cliques using the Bron-Kerbosch algorithm [41], similar to Dercksen et al. [23]. Due to the high memory requirement of 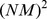 in the worst case and the exponential running time of the Bron-Kerbosch algorithm, the DCG graph should contain fewer than 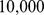 nodes. In practice, the number of vertices in 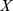 and 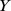 should be less than 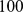. This is often much fewer than the number of endpoints of microtubule centerlines in tomograms. We therefore use a heuristic to reduce the number of points: First, we sort all endpoints in descending order of angle of the line with the cutting plane of the section. Then, we pick the first 50 to 100 endpoints in each list. The microtubules that are most perpendicular to the plane of sections are expected to be easiest to match in the initial alignment. Furthermore, we only consider a clique if its size is at least 10 to 30% of the number of nodes in the smaller set. Also, we only consider the first 1000 cliques computed by the Bron-Kerbosch algorithm.

From the resulting endpoint correspondences, we compute an optimal (in a least-square sense) rigid alignment of the form 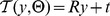 using singular value decomposition (SVD) as described by Umeyama [42] and Myronenko and Song [43]. To compute this alignment, we only take into account the endpoint positions. Line orientations could probably be incorporated by formulating the transformations with dual quaternions as described by Walker et al. [44]. The method described above, however, seems sufficient to compute a coarse initial alignment for sufficiently straight lines.

### Fine alignment

The initial alignment considered only a subset of endpoints. We next describe how to compute a fine alignment from all endpoints for a linear and an elastic transformation model. The main text summarizes the methods. A detailed derivation of equations and the precise algorithms are given in the Supporting Information. Our fine alignment algorithms build upon the work of Myronenko and Song [28]. After briefly recapitulating their approach, we describe our extension to incorporate line orientations in the formulation. A basic understanding of probability distributions and statistical learning is assumed. For a practical introduction to statistics, see, for example, Dekking et al. [45], or Bishop et al. [46] for a more thorough treatment.

#### General methodology

The general idea is to formulate a probabilistic model and find the transformation 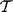 that maximizes the likelihood of the data. The probability of a point 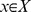 being a match for 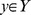 is modeled as a joint distribution of a 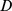-dimensional continuous random variable 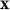 and a discrete random variable 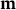 with states 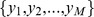


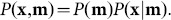
(1)For two sets of two-dimensional points, 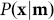 will be a Gaussian 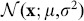 located at the transformed 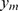. 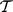 will be a transformation 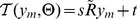, where 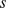 is a uniform scaling factor, 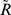 is a two-by-two rotation matrix, and 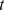 is a translation vector. Written out, 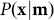 then reads:
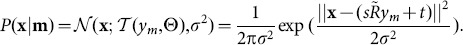
(2)The prior 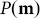 is uniformly distributed as 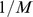 since we have no measure of certainty of the 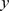
s and therefore assume that each is equally valid.

**Figure 4:**
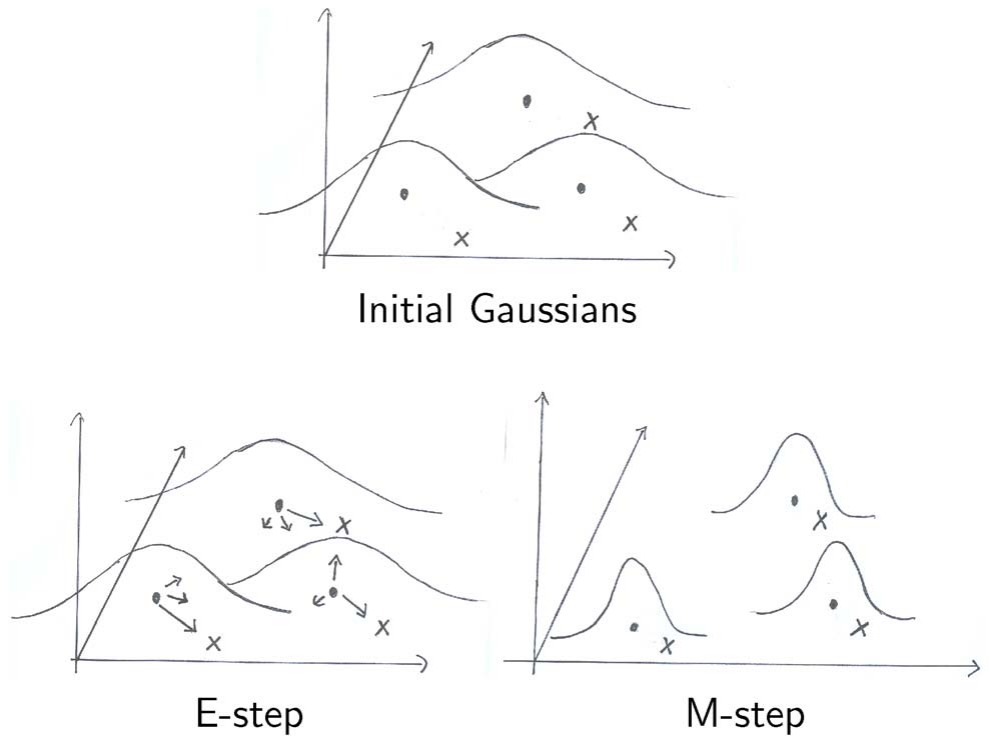
Illustration of the expectation maximization algorithm for a Gaussian mixture model. Dots represent the centroids of the Gaussians which are located at 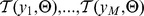. Crosses represent the points in *X*. E-step: Arrows indicate values of the posterior for each point in *Y*. M-step: Steps towards convergence move the points closer and reduce the width of the Gaussians. Upon convergence, corresponding points should be close to each other and the Gaussian should be sharply peaked.

To find the optimal parameters 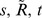 and 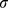, Myronenko and Song use the expectation maximization algorithm. Two steps are performed iteratively to compute optimal transformation parameters (illustrated in Figure 4). First, the posterior 
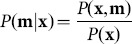
 is computed in the expectation step (E-step). Here, 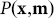 is given by Equation 1 and 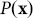 can be obtained from 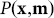 by marginalizing out 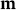. The posterior for each pair 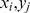 can be seen as a weight that indicates how much 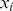 pulls 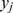 closer. Second, transformation and distribution parameters are updated by minimizing the expectation of the complete negative log-likelihood 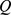 with respect to 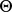 and the parameters of the distribution. This step is called the maximization step (M-step). 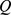 is given by
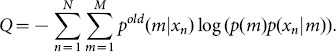
(3)The first term, 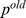 is the posterior 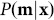 which was computed in the E-step and is kept fixed during optimization. 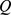 is minimized with respect to the new transformation parameters 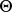 and the parameters of the distribution. In case 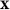 is distributed as a Gaussian and 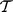 is the transformation from above, we would minimize 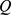 with respect to 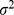 and 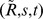. The detailed algorithm is given in Myronenko and Song [28].

The expectation maximization algorithm always converges to a local minimum (see Bishop et al. [46]). To adapt this algorithm for lines we 1) define a distribution for the lines, 2) define a transformation model and 3) find a way to compute 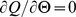 for each parameter 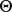 in the M-step.

#### Linear alignment

For the linear alignment, we model the transformation of the line orientations as a rotation matrix 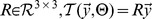. Assuming that all endpoints lie in a plane, we restrict the rotation to be around an axis that is perpendicular to the cutting plane, so that
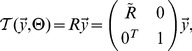
(4)where 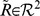 is a two-dimensional rotation matrix. We model the transformations of the endpoint positions as 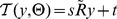, where 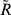 is the same two-dimensional rotation matrix, 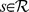 is a uniform scaling parameter, and 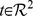 is a translation vector.

The *algorithm for linear alignment from orientation* uses only the line orientations during expectation maximization, from which only the rotation 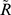 can be directly computed. We introduce two more random variables 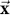 (continuous) and 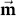 (discrete with states 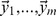) and describe the joint distribution of the orientations by
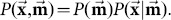
(5)Because 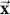 is periodic, we assume that 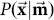 is distributed as a Fisher-Mises distribution (see Mardia [47]):
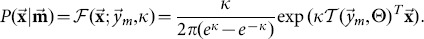
(6)


**Figure 5:**
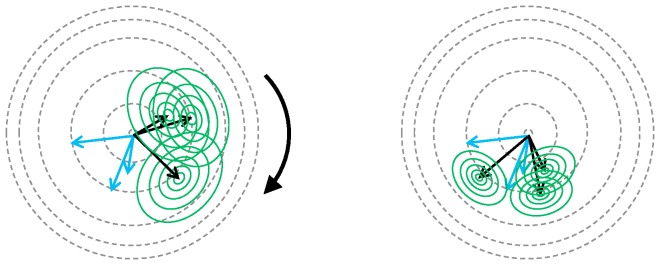
Illustration of the expectation maximization algorithm for periodic variables. The dashed circles illustrate the unit sphere. Arrows indicate the orientation unit vectors projected onto the plane (blue: 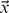, black: 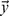). The green circles depict values of the Fisher-Mises distribution with centers located at the arrow tips. In the M-step, a rotation around the center is computed that moves the arrow tips closer. Simultaneously, the concentration parameter 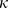 is updated and the width of the Fisher-Mises distribution is reduced. Left: before M-step. Right: after M-step.

The Fisher-Mises distribution is equivalent to a Gaussian distribution on a sphere (illustrated in Figure 5). The prior 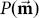 is uniformly distributed as 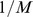 as in the work of Myronenko and Song [28].

To obtain the optimal rotation in the M-step, we write down the expectation of the negative complete log-likelihood function (Equation 3) and minimize 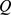 with respect to 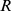 and 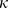. We give details of this derivation in the Supporting Information. In brief, the optimal rotation can be obtained with a singular value decomposition (see Umeyama [42] and Myronenko and Song [43]). 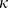 cannot be obtained in closed form, but it can be computed with Newton's method.

Even though we did not consider the endpoint positions during expectation maximization, we can apply the final rotation to the positions and compute a translation vector from all potential pairs weighted by the posterior (see Supporting Information). The complete algorithm is summarized in Figure S1.

The *algorithm for linear alignment from position and orientation* considers both endpoint positions and line orientations and uses the full linear transformation model as introduced in Equation 4 and below. We define a joint distribution 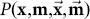 that factorizes as follows:
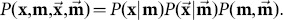
(7)

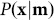
 and 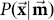 are defined by Equation 2 and 6 respectively. We define the prior 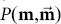 by
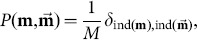
(8)where 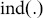 denotes the index operator, which returns the index of the current assignment and 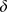 is the Kronecker delta which is 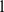 if 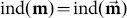 and 0 otherwise. We chose this prior because we know the pairs 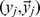, and pairs with mixed indices 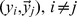 do not occur.

We can again write out 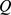 for this distribution and the transformation model and then solve for the 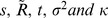 that minimize 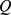 in the M-step. However, we do not have a closed-form solution for the optimal parameters. Instead, we minimize 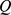 numerically. We give a derivation of the update equations and the first and second order derivatives necessary for the numerical optimization in the Supporting Information. For optimization, we use the library IPOpt [48]. The algorithm is summarized in Figure S2.

#### Elastic alignment

A linear transformation cannot correct distortions, which usually appear in electron tomograms. To correct such distortions, we apply an elastic alignment.

For the *algorithm for elastic alignment*, we model the transformation as individual translation vectors 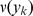 that modify the positions such that 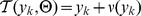. The formulation closely follows Myronenko and Song [28]. They obtain the translation vectors by minimizing the regularized 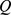 function with calculus of variations. The resulting vectors 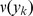 are the product of an 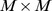 smoothing matrix 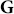 and an 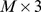 matrix 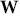, that is 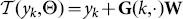, where 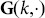 denotes the 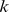
th row of 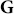. Here the transformation parameters 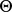 are the entries in the matrix 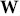, whereas 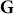 is initialized once as 
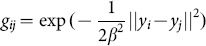
 and stays fixed. Myronenko and Song call the algorithm for computing the optimal 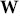 the coherent point drift algorithm (CPD) for non-rigid point set registration.

Incorporating orientation into the described transformation model is not obvious, because the rotation that should be applied to the orientations is not apparent from the translation vectors 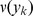. In practice, however, we can assume that orientations are fixed, because we performed a linear alignment before. It is therefore reasonable to assume that the remaining elastic deformation contains negligible rotation. With this assumption, the orientations only influence the result via the posterior 
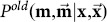
. 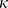 can be updated in each iteration again with Newton's method. See details in the Supporting Information. The algorithm is summarized in Figure S3.

We use the moving least squares algorithm as described by Schaefer et al. [49] to interpolate the deformation and apply it to the tomograms. The displacements vectors 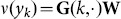 define the landmarks for moving least squares.

### Endpoint matching using Markov random field

**Figure 6:**
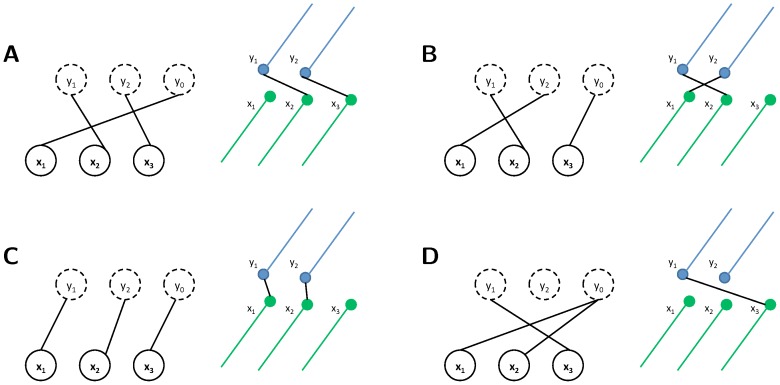
Examples of matching configurations. Centerlines from two consecutive sections are depicted in blue and green. The random variables 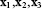 are illustrated on the left of each panel. Connections to 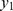 and 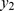 indicate assignments to endpoints in the neighboring section. Connections to 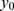 indicate assignments to the placeholder. A and C: consistent assignments. C should be preferred because the corresponding points are closer. B and D: assignments that should be penalized.

The previously defined distribution describe the probability that endpoints correspond. However, we cannot simply assign each endpoint in 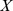 to its most probable counterpart in 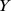, because assignments might conflict and the final pairs might not be unique. A common approach to finding unambiguously corresponding pairs is to compute a maximum weighted matching (MWM) on a bipartite graph (see Kuhn [29]). Furthermore, neighboring assignments might influence each other. For example, we would expect that the assignment of endpoints in Figure 6A is preferable to the assignment in Figure 6B, because remaining deformations of the data should be coherent in a neighborhood.

To find a matching of the endpoints, we use a Probabilistic Graphical Model (PGM) (see Koller and Friedman [30] for a comprehensive introduction) to model the influence of neighboring assignment on a pairing decision. Here, endpoints in 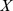 are represented by discrete random variables 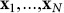, each of which can have 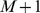 states: either 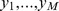 to indicate the matching endpoint in 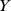 or the placeholder 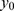 to indicate that there is no match. Each 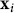 must be assigned with the constraint that two 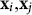 cannot be assigned to the same 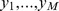. Multiple assignments to the placeholder 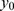 are explicitly allowed. Figure 6 illustrates possible states for a joint distribution of three variables.

To find a good assignment, we first define the joint distribution 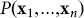 and then determine the joint assignment that yields the largest probability. 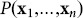 is defined by a Gibbs distribution (Koller and Friedman [30])
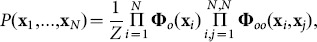
(9)each state of which is a valid matching. 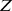 ensures that 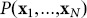 is a valid probability distribution. The 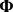
's are called *factors*. Since the 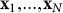 are discrete random variables with values from a finite set, the factors are discrete tables with entries that can be though of as weights that represent our beliefs about a particular assignment. The *singleton factors* 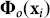 contain information on how likely 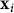 and each 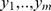 or 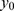 match if no further information is available. The *pair factors* 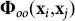 represent beliefs about the joint assignment 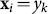 and 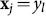. We can use the pair factors to model, for example, the mutual exclusiveness constraint of the assignments.

#### Factor values for matching microtubule endpoints

**Figure 7:**
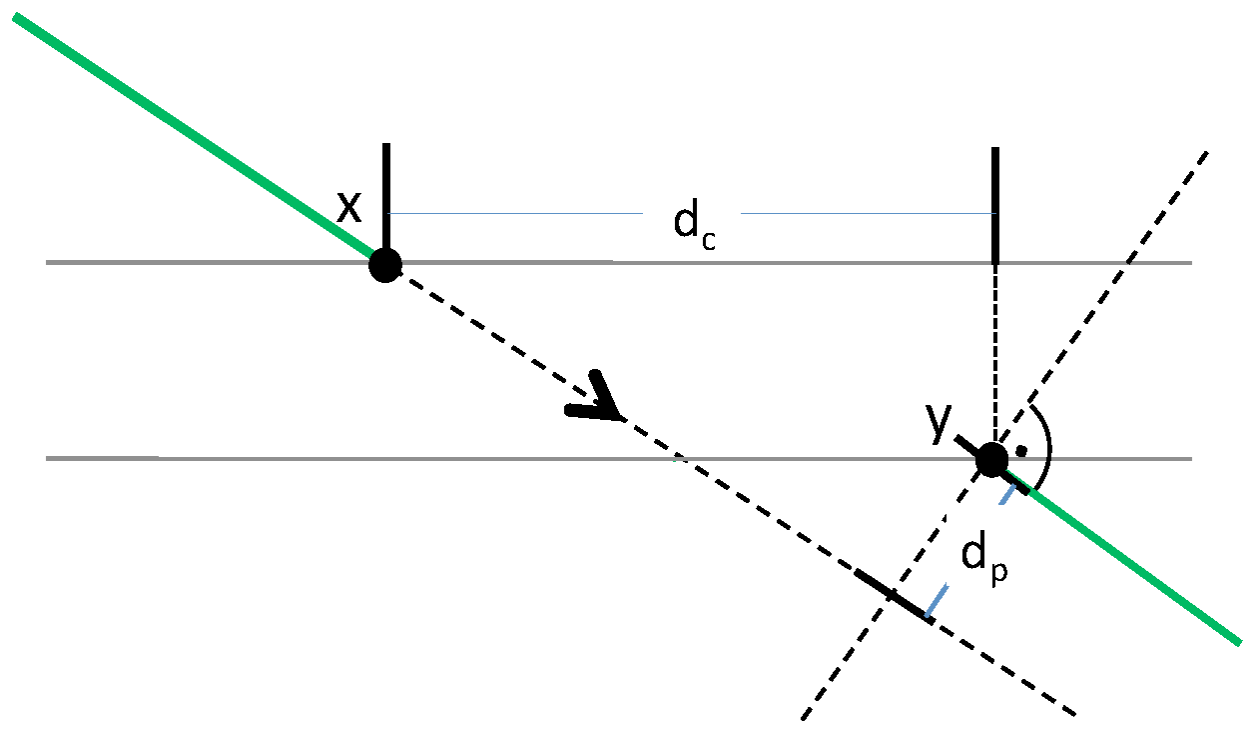
Endpoint distances. 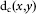
 is the direct distance, which is computed as the horizontal distance between 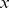 and 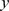. 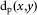 is the projected distance, which is computed by projecting 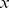 along the line orientation at 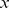 onto a plane through 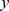 that is perpendicular to the orientation at 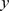.

We use three types of information to fill the factors 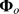 in Equation 9. First, we assume that microtubules are rather straight and, therefore, expect the angle 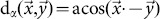 to be small. Second, we expect that the positions of matching endpoints are close and, therefore, compute the *direct distance* 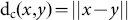. 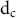 measures the distance on a plane, which we assume all endpoints to lie on (see Figure 7). Third, we expect that the straight extensions of corresponding lines should meet. To measure how close they get, we define the *projected distance* 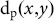 as the distance of 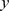 to the intersection of the line through 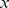 and a plane at 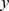 that is normal to 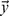 (see Figure 7). Each of the three 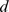
's is used in an exponential distribution with parameters 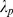, 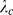, 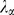, for example 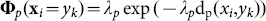 (see also Amat et al. [33]). For assignments to the placeholder 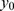, we use placeholder distances 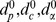. The individual factors are multiplied to define the singleton factors
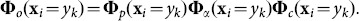
(10)


To account for joint assignments, we fill the pair factors as in Amat et al. [33] by considering the pair of displacement vectors 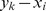, 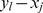 induced by a joint assignment 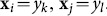. We define 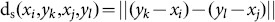. If 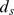 is large, then the displacement vectors disagree. For example, 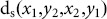 in Figure 6B would be larger than 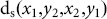 in Figure 6C, because the connections cross and the displacement vectors are farther apart. We want to prevent such joint assignments and, therefore, penalize large values of 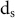 by filling 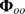 with
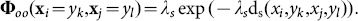
(11)For assignments that involve the placeholder 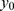, we use the placeholder distance 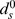. Furthermore, we set entries to 0 if 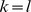 to account for the mutual exclusiveness constraint (see Koller and Friedman [30]). To reduce the number of factors that have non-zero entries, we introduce thresholds that restrict assignments to include only close-by points 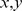, specifically 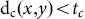, 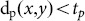 and 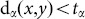 We then omit all pair factors for variables that cannot be assigned to the same 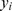. In practice, this causes the distribution to decompose into several independent joint distributions of subsets of the random variables that can be solved independently.

#### Maximum a posterior assignment

Each valid matching is a single joint assignment in the distribution 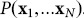. The goal now is to compute the maximum a posteriori (MAP) assignment 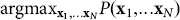, the joint assignment with the greatest probability. Computing a MAP assignment is not generally feasible, because the problem is NP-hard.

**Figure 8:**
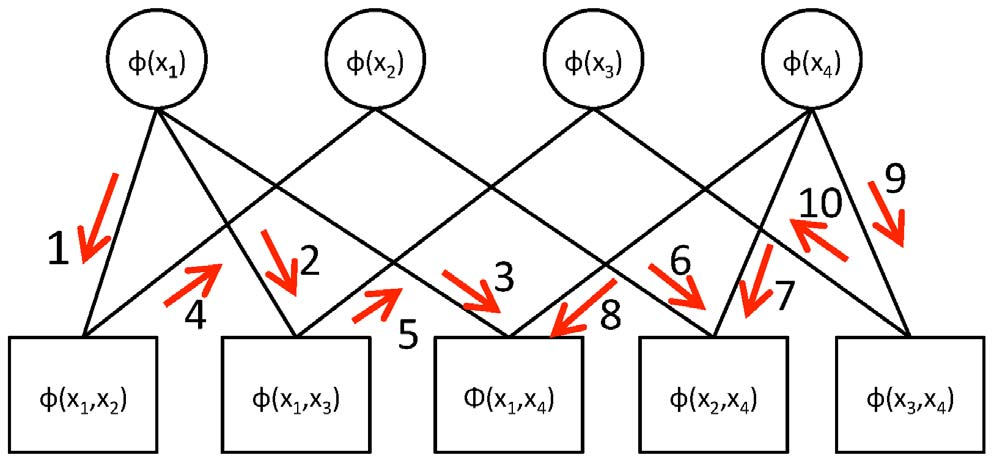
Illustration of a factor graph with message passing. Circles depict singleton factors. Squares depict pair factors. Red arrows and numbers indicate a possible message passing schedule.

We use an approximation technique called belief propagation on a graph representation called a factor graph (see Koller and Friedman [30], Amat et al. [33] and Kschischang et al. [50]). Briefly, each node in a factor graph represents one of the factors as depicted in Figure 8. Nodes are connected if their factors share a variable. Messages that express the belief about a MAP assignment of a variable are passed between the nodes in a particular order (red arrows and numbers in Figure 8). Each node uses incoming messages and its own factor value to compute its own belief. This is in turn passed on to the next nodes. For example, in Figure 8, the node representing the pair factor for 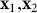 uses message 1 to compute a belief about the assignment of 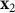 and passes this belief to the node representing the singleton factor for 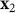 in message 4. The message passing finishes if all messages agree. The MAP assignment can be read from the messages in the graph after convergence.

Unfortunately, convergence is not guaranteed. Success depends on the underlying model. If two assignments conflict, messages with different beliefs about MAP assignments will be passed back and forth and the algorithm will not converge. This oscillatory behavior is often a local problem of a network (see Koller and Friedman [30], 401 pp, 570 pp). To achieve convergence, we identify the endpoints that cause oscillation. We then point an expert to these endpoints and ask to provide a manual assignment. The opinion on a particular assignment is incorporated into the statistical model by setting the chosen assignment in all associated factors to 1 and all other entries to 0. We then run belief propagation again to check if all conflicts have been solved and convergence is achieved. If not, the expert is asked for more assignments.

To identify nodes that cause oscillation, we compute the disagreement of two messages 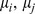 about variable 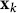 as the 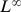 norm of the difference vector, 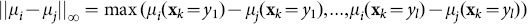, where 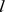 is the number of possible states of 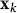. We collect for each variable each incoming message for all factors that have the variable. For example, in Figure 8, we would collect messages 1, 2 and 3 for variable 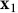, messages 4 and 6 for variable 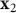 and so on. We compute the disagreement for each pair of messages. We define the maximum disagreement for each variable as the maximum of the computed differences. For each independent network in the factor graph, the variable whose messages disagree most is then shown to the expert. We refer to these endpoints as *critical nodes*.

#### Parameters

The PGM algorithm for point matching has 11 parameters: the four weights 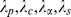, the three thresholds 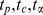, and the four placeholder distances 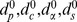. We determine the thresholds and the placeholder distances from their corresponding 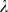
's by choosing a single placeholder significance parameter 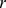 in the range 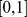. The placeholder distances are then computed such that the cumulative distribution function of the exponential distribution 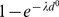 reaches 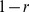. Thus 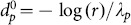 and equivalently for 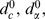 and 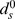. We use the same values for the thresholds: 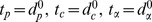. Intuitively, 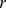 controls how much of the cumulative distribution function is ignored by ignoring assignments to real endpoints above a certain distance. We used 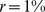 in all our experiments.

We determine reasonable choices for the weights 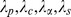 with a maximum-likelihood estimate using the ground truth. To do so, we consider Equation 9 as being the likelihood function for the distribution when only one sample was seen. Because the maximum-likelihood estimate for the parameter of an exponential distribution is directly related to the mean of the data, we can compute 
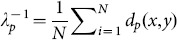
 and equivalently for 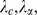 and 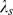. Figure S12 illustrates the choice of the parameters for our samples.

The parameter choice influences two aspects of the result. First, while manual input about assignments necessarily enforces convergence of belief propagation after a finite number of steps, we cannot know in advance how many assignments and iterations are needed. To measure it, we experimentally tested the procedure on the evaluation samples described. We iteratively ran belief propagation (5 passes) and assigned the endpoints that caused oscillation automatically by reading the assignments from the ground truth until belief propagation converged (see Results).

Second, the parameter choice might influence the quality of the result. We distinguish three types of errors: 1) False negatives (FN): A pair assignment in the ground truth is missing in the automatically computed matching. 2) False positives (FP): A pair was computed that is not in the ground truth. 3) Disagreements (D): An endpoint was matched differently in the ground truth and the automatic assignment. We also measure how many pairs were assigned correctly, that is, the true positives (TP). To estimate the sensitivity of the parameters 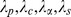, we varied their value around the maximum-likelihood estimate. For each value, we ran belief propagation and assured convergence using the ground truths as described before. We measured the number of iterations, the number of manual assignments, and the number of FN, FP and D resulting from the computed matching. Furthermore, to get an estimate on the error rate, we provide the precision P = 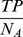, recall R = 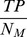, and fraction of disagreeing matchings DIS = 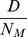. 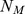 here is the number of pairs in the ground truth and 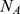 the number of pairs in the automatic matching (see Results).

#### Implementation

We use libDai [51] to run belief propagation on the network with the scheduling proposed by Elidan et al. [52]. The graphical user interface has been implemented in Amira [40].

### Ground Truth for Evaluation

To prepare a ground truth, we applied the described computational methods. The programmer assigned evidence as she saw fit to the PGM point matching. The result was then verified and corrected by experts in biology using a graphical user interface (see Figure S5). The user interface supports inspection of the line geometry in a perspective view and in a separate view together with an oblique image slice. Both views can be interactively changed at any time. Different sections are indicated by colors. To support the verification process, the user interface automatically navigates to endpoints and restricts the perspective view to show only neighboring lines and endpoints. The experts made decisions primarily based on such closeup views. They inspected the three-dimensional situation by interactively changing the viewing direction to verify that connections of neighboring lines were consistent. The experts felt that they could make reasonable decisions. They systematically verified all endpoints close to the section boundaries and corrected wrong connections or added missing connections. It was sometimes helpful to verify lines also in an overview that shows larger parts of a section. The experts did not add new lines that seemed to be completely missing in neighboring sections. We accepted missing lines, because the primary purpose of the evaluation was to determine whether the result of automatic alignment and matching agrees with an expert's opinion, and some lines are expected to be missing in tracings from individual sections.

## Results

**Figure 9:**
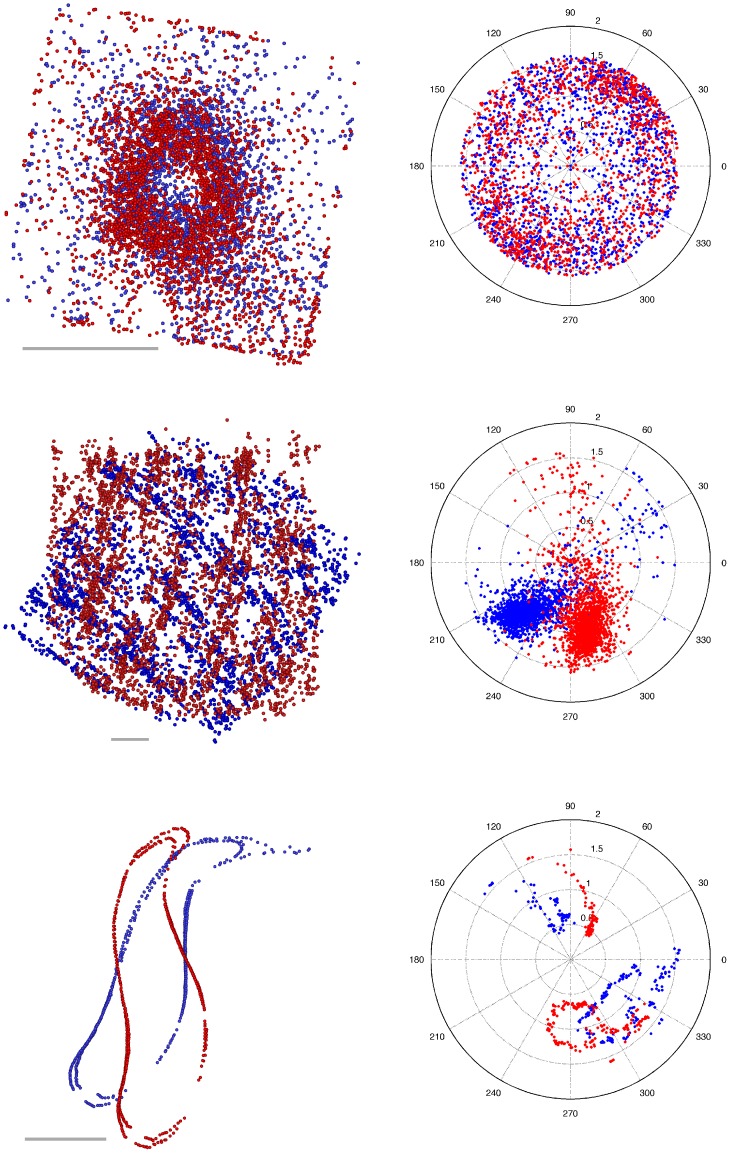
Endpoint positions and orientations of the evaluation samples. Endpoint positions and orientations on the facing boundaries of two consecutive unaligned sections. The different sections are indicated by color. Left: endpoint positions (view from top). Right: line orientations plotted in polar coordinates. Top: *C. elegans*. Middle: *X. laevis*. Bottom: *T. brucei*. Scale bars 2 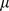
m.

To understand the performance of the computational methods, we tested them on samples of different characteristics. We used more than 50 pairs of sections from *C. elegans* mitotic spindles for general testing. See Figure 1 for an example of a stitched stack of 20 sections. The *C. elegans* samples are relatively small in size (the centrosome is less than 5 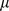
m in diameter). Microtubules extend from a centrosomal region that can be covered with a single frame tomogram. The samples contain approximately 1500 lines per section. Endpoints are spread in a ring-like structure around the microtubule organizing center and orientations are distributed homogeneously (Figure 9 top). We limited detailed testing to three pairs of consecutive sections, for which we created a ground truth. Two experts independently verified and corrected the computed matching for the whole section boundaries. One of the ground truth samples is displayed in Figure S6.

We also used a stack of three sections of a *X. laevis* meiotic spindle. The sample is larger, and tomogram montaging was necessary. Each section contains approximately 3500 lines. Finding a transformation is hard due to deformations. Establishing endpoint correspondence is difficult, because microtubules are organized in dense bundles. Figure 9 (middle) depicts endpoint positions and line orientations for a small area of these sections. Line orientations are clustered. Unlike in the *C. elegans* centrosome, most microtubules in the *X. laevis* spindle have a similar orientation. Since the sections are huge, we created a ground truth only for a subregion. Manual verification and correction was preformed on a region that contains approximately 1000 lines per section (see Figure S7). The subregion covers a relevant part of the tomogram and should contain a variety of typical configurations, which were indeed observed by the expert during verification. We did not, however, perform additional tests that the region is representative.

Furthermore, we used a sample of the sub-pellicular microtubule array of *T. brucei*, which is a sheet of parallel microtubules underlying the plasma membrane (reviewed in Gull [53], Farr and Gull [54]). Figures 9 (bottom) depicts endpoint positions and line orientations of two consecutive sections. Tomogram montaging was used. Each section contains 200–400 microtubules. This sample is challenging, because microtubules are arranged in sheets. No ground truth was prepared for the *T. brucei* data.

The running times of all algorithms were below 2 minutes per ground truth section pair (single threaded optimized code, CPU AMD Opteron 6174).

### Initial alignment

**Figure 10:**
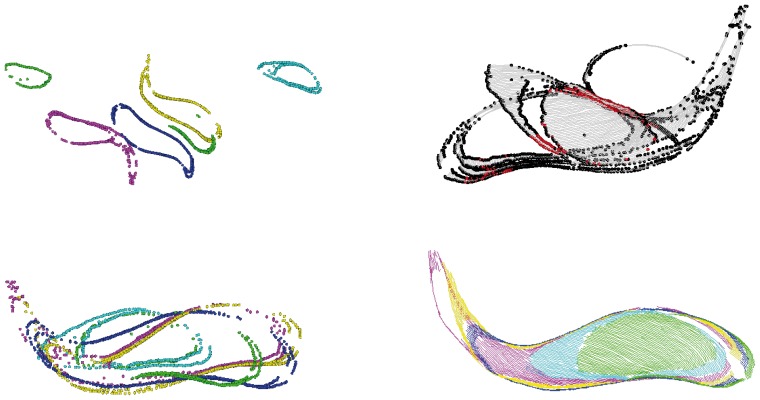
Alignment of *T. brucei*. The view is from the top. Unless stated otherwise, colors indicate 6 different sections. The stacking order is best seen in the final result (bottom right) at the right side: green, cyan, purple, yellow, blue, green. The length of the microtubule array is approximately 8
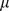
m. Top left: initial position of unaligned sections. Only endpoints are displayed. Top right: The result after applying the initial alignment algorithm. Endpoints in all sections are depicted in black; lines in gray. The subset of endpoints that were used for computing the transformation are highlighted in red. Some pairs of sections are reasonably aligned. Other pairs are unaligned. Bottom left: endpoints after applying our algorithm for linear alignment from orientation (Figure S1). All sections are reasonably rotated. Bottom right: result after applying our algorithm for linear alignment from position and orientation (Figure S2). Endpoints and lines are displayed, both colored by section. All sections are reasonably aligned.

Our initial alignment algorithm worked reliably for *C. elegans* tomograms and reasonably well for *X. laevis* tomograms, but it failed on *T. brucei* tomograms. A reasonable initial alignment was found for more than 50 pairs *C. elegans* sections. We used 50 endpoints in each section to build the DCG. In approximately 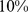 of the cases, we had to scale one of the two sections between 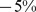 and 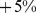 to obtain a reasonable result. We tried several uniform global scalings until we found a reasonable result. For four *X. laevis* samples (each contained approximately 3500 lines), we had to pick at least 100 endpoints from each section, restrict the size of the cliques in the DCG to 10 endpoints, and test different scalings to obtain a rough alignment. For *T. brucei*, we rotated the sections to simulate completely unaligned tomograms (Figure 10, top left). The algorithm failed to compute an initial transformation. No matching points could be identified by comparing angles of line orientations. The reason probably is that all lines are organized in a regular pattern more or less in parallel with a fixed spacing (Lacomble et al. [9]).

### Fine alignment

#### Robustness of linear alignment

**Figure 11:**
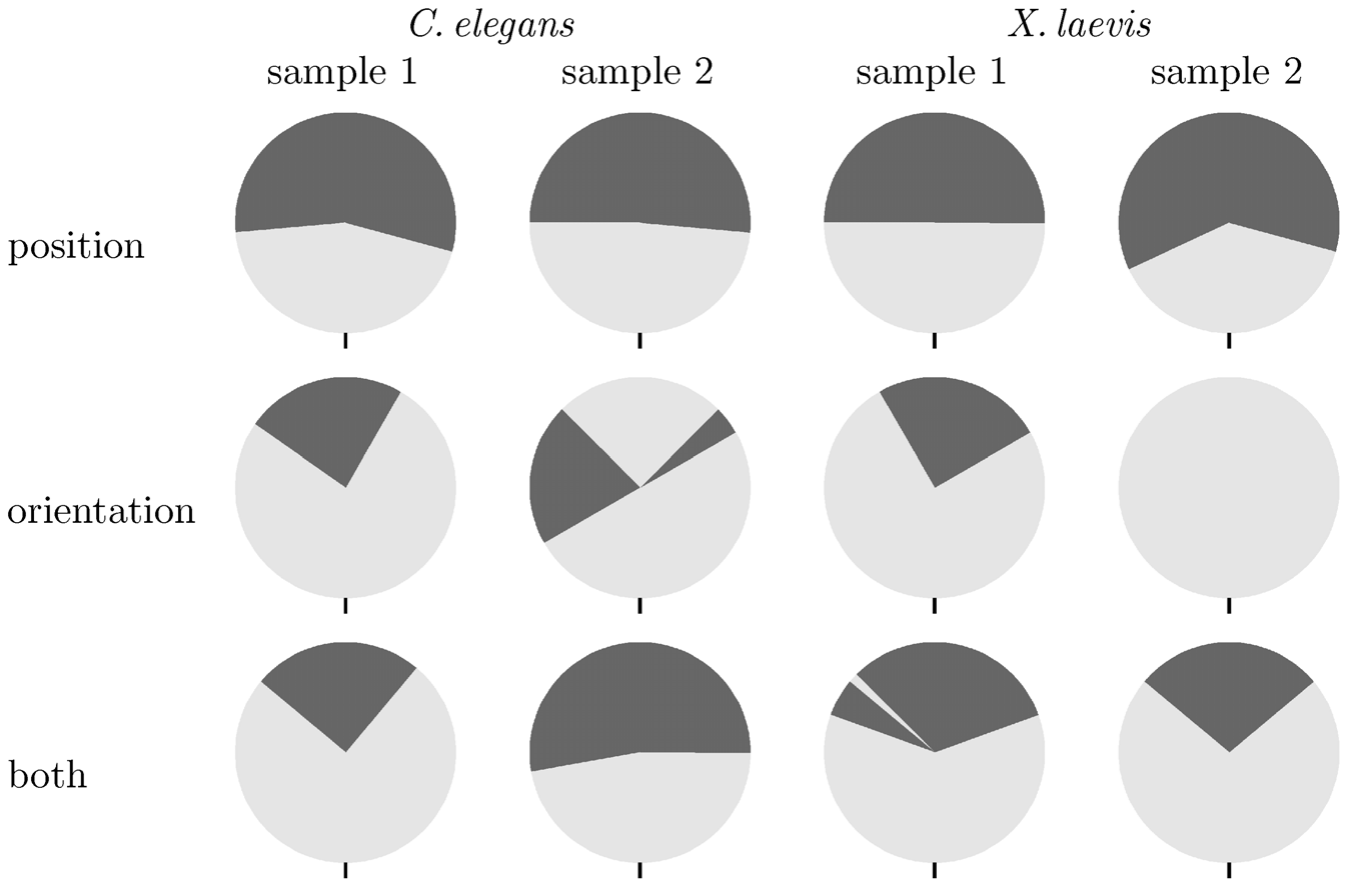
Stability of alignment. We rotated one section of a pair of correctly aligned sections for several samples over a range of 360° at 5° steps, as depicted by the circles, and tested whether the algorithms recovered the originally correct orientation, which is indicated by a vertical bar at the bottom of each circle. Dark gray indicates failure of the algorithm to recover the original rotation angle. Light gray indicates that the algorithm discovered the correct rotation. All algorithms found correct alignments for small rotations (light gray area in bottom half of each circle). The algorithm for linear alignment from orientation was most successful (largest light gray areas in middle row). Top: Rigid point set registration algorithm by Myronenko and Song (Figure 2 in [28]). Middle: Algorithm for linear alignment from orientation (Figure S1). Bottom: Algorithm for linear alignment from position and orientation (Figure S2).

We tested robustness of the linear alignment and found that our algorithm for linear alignment from orientation (Figure S1) and our algorithm for linear alignment from position and orientation (Figure S2) seem to be a bit more robust than the original rigid point set registration algorithm by Myronenko and Song (Figure 2 in [28]). To test robustness, we rotated one section of a pair of correctly aligned sections for several samples over a range of 360° in 5° steps and tested whether the three algorithms were able to rediscover the original rotation. We used two samples from the *C. elegans* ground truth and two randomly chosen subregions from the *X. laevis* samples. Results are summarized in Figure 11. All algorithms found correct alignments for small rotations. The algorithm for linear alignment from orientation found the original rotation in more cases than the other algorithms. Upon convergence, 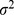 was 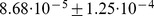 (mean 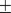 standard deviation) when the rotation angle was correctly computed and 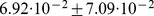 when the angle was incorrect. 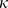 was 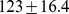 for correct and 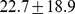 for incorrect results. Correct results can be clearly distinguished from incorrect results by inspecting 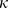 and 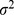.

We also tested the algorithms on the *T. brucei* sample (Figure 10) and observed that satisfactory results could only be achieved by applying the linear alignment in two steps. The original rigid point set registration algorithm by Myronenko and Song (Figure 2 in [28]) and our algorithm for linear alignment from position and orientation (Figure S2) both failed to compute a correct result from the initial alignment (Figure 10, top right). A combination of the following two steps, however, yielded a good result: By using our algorithm for linear alignment from orientation (Figure S1) first, a reasonable rotation could be computed (Figure 10, bottom left). In the second step, a refined alignment could then be computed (Figure 10, bottom right) with our algorithm for linear alignment from position and orientation (Figure S2).

#### Displacements during elastic alignment

**Figure 12:**
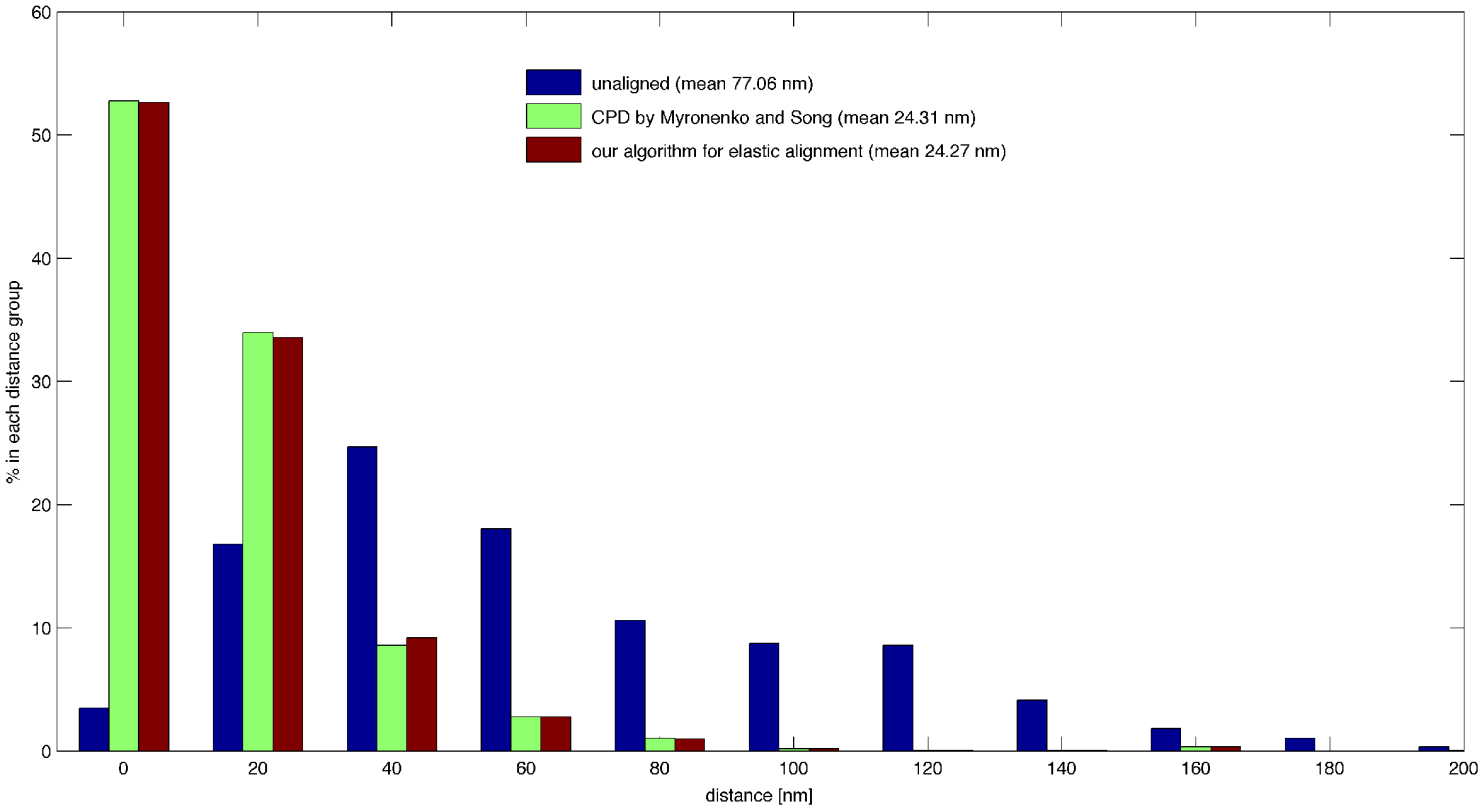
Distances before and after alignment. Histogram of the distances between corresponding endpoints for the *X. laevis* ground truth before and after applying the non-rigid point set registration algorithm by Myronenko and Song (Figure 4 in [28]) and our algorithm for elastic alignment.

The original CPD algorithm (Non-rigid point set registration algorithm, Figure 4 in [28]) and our algorithm for elastic alignment (Figure S3) compute similar displacements. We compared the two algorithms by measuring the distances of the manually connected endpoints before and after applying the transformation to the *X. laevis* ground truth. Figure 12 shows the histograms of the distances. Both methods performed similarly. The original CPD, however, needed roughly 1.5 as many iterations. We measured similar factors for other samples.

#### Limitations

The algorithm for elastic alignment yielded a reasonable result for most parts of the *T. brucei* sample. However, some parts were not aligned properly, see Figure S8. Here, the segmented lines were often too long or too short, and the assumption that point displacements are coherent in a small neighborhood was violated. The proposed deformation model failed to handle such cases.

Precisely locating the relevant part of a sample under an electron microscope is difficult, and serial tomograms, therefore, might be shifted to each other. If the shift is large, only a small portion of the lines in one section has a corresponding counterpart in the next section. To test whether the algorithms handles small overlaps, we cut two regions with little overlap out of a *X. laevis* (Figure S9) and applied the alignment algorithms. None of the algorithms yielded a reasonable result.

### Endpoint matching

#### Parameters of the probabilistic graphical model

**Figure 13:**
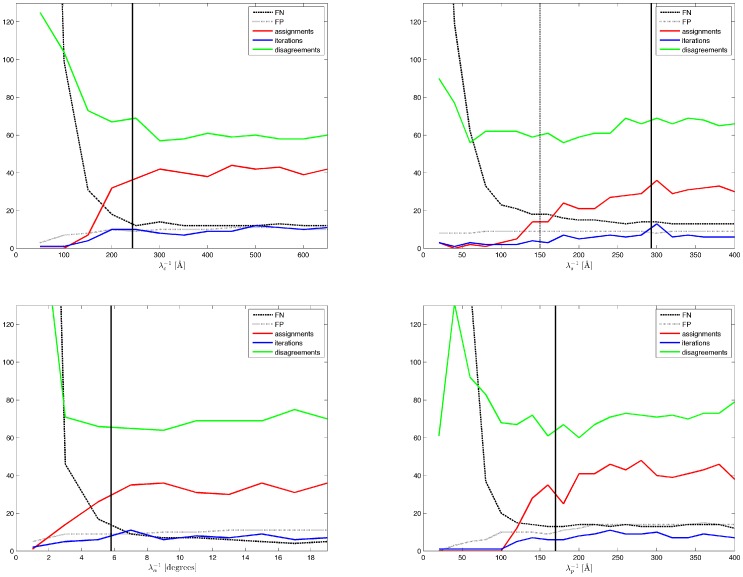
Matching performance over parameter variations. Displayed are false negatives (FN), false positives (FP), number of manual assignments, number of iterations, and number of disagreements when varying matching parameters around their maximum-likelihood estimates (indicated by solid vertical line) for the *X. laevis* sample: top left: by direct position distance parameter 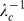; top right: by shift difference parameter 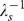 (the dashed vertical line indicates the value that we chose instead of the maximum-likelihood estimate); bottom left: by angle difference parameter 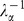; bottom right: by projected distance parameter 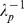.

The described maximum-likelihood estimate delivers reasonable parameters, and matching results are relatively insensitive to parameter variations. We used the ground truth of the *X. laevis* sample to estimate parameters: After linear alignment but before elastic alignment, parameters were estimated as 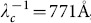, 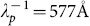, 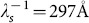, and 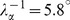. After applying the algorithm for elastic alignment, parameters were estimated as 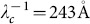, 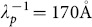, 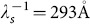, and 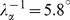 (see Figure S12). The smaller values for 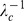 and 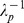 confirm that the elastic alignment moved corresponding endpoints closer. To fine-tune the parameters and test their sensitivity, we varied each parameter separately around its maximum-likelihood estimate and measured the performance of the PGM matching on the *X. laevis* sample to which the algorithm for elastic alignment had been applied (see Figure 13). The performance was stable over a wide parameter range. We also estimated the parameters from the *C. elegans* samples to which the algorithm for elastic alignment had been applied: 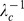 was in the interval 
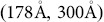
, 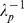 was in the interval 
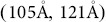
, 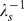 was in the interval 
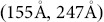
, and 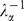 was in the interval 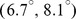.

We used the maximum-likelihood estimates that were computed from the *X. laevis* sample after elastic alignment for further experiments, except for the pair shift parameter 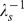, which we set to 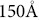, because fewer manual corrections were required at this value (see Figure 13). We saw no reason to choose different parameters for individual specimens, because the matching was stable over a wide parameter range; the estimates from the *C. elegans* samples were in a similar range as the estimates from *X. laevis*; and the matching quality later turned out to be good for *C. elegans*.

#### Quality of matching

We compared the result of the PGM matching with the ground truth samples. We used a placeholder significance of 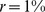 in all experiments. Figure S10 displays typical differences that we observed for the *X. laevis* sample. When the PGM matching disagreed with the ground truth, the PGM's choice often seemed reasonable, too. Some situation with disagreement seemed inherently difficult to decide. In some situations, this might be caused by lines that are missing in one section. Figure S11 displays typical differences that we observed for the *C. elegans* samples. We observed far fewer disagreements than for the *X. laevis* sample. The reason probably is that lines are oriented more arbitrarily. They do not form bundles, and ambiguities seem less likely. We observed a few additional or missing lines in the PGM matching.

We found that the result of the PGM matching clearly outperforms an MWM for the three sections of the *X. laevis* spindle. Table 1 contains a quantitative comparison. We first set the parameters to their maximum-likelihood estimates from above and computed the matching for the sample to which the algorithm for elastic alignment had been applied (Table 1, configuration X1). To examine the effect of parameter variations, we ran the matching again with parameters set to twice their maximum-likelihood estimate (Table 1, configuration X2). To test stability of the PGM with respect to alignment quality, we ran the matching on the *X. laevis* sample to which the algorithm for linear alignment from position and orientation had been applied but not the algorithm for elastic alignment. We used the maximum-likelihood parameters as estimated above for this situation: 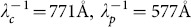 and 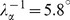 except for the pairwise shift 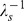, which we again set to 150 (Table 1, configuration X3). The results indicate that the PGM approach is unaffected by parameter variations and imperfect alignment in terms of precision (0.96), recall (0.95) and fraction of disagreeing matches (0.04); see Methods above for definition of the quality measures. MWM performed worse, with precision and recall dropping below 0.9 for parameters at twice the maximum-likelihood estimate and below 0.65 without elastic alignment. In all cases, only a small fraction of endpoints (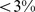) had to be assigned manually to achieve convergence. It took an expert 1 h per 100 nodes to provide assignments. Checking unconnected lines after the final matching took 1 h per 500 endpoints.

**Table 1:** Comparison of probabilistic matching (PGM) and maximum weighted matching (MWM).

*X. laevis*							
	PGM				MWM		
	P	R	DIS	Assign.	P	R	DIS
X1	0.956	0.951	0.038	4	0.901	0.904	0.088
X2	0.950	0.953	0.039	8	0.851	0.874	0.121
X3	0.956	0.948	0.038	19	0.609	0.634	0.349

*C. elegans*							
	PGM				MWM		
	P	R	DIS	Assign.	P	R	DIS
	0.946	0.975	0.01	0	0.934	0.97	0.018

Precision P: ratio of correct pairs to total pairs found by the matching. Recall R: ratio of correct pairs to total pairs in the ground truth. Disagreement DIS: ratio of automatic matchings that disagree with the ground truth. Assign.: number of manual assignments. *X. laevis*: X1: after elastic alignment; PGM parameters 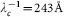, 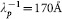, 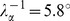, and 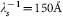. X2: after elastic alignment; PGM parameters 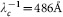, 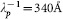, 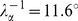, and 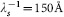. X3: after linear alignment, but before elastic alignment; PGM parameters 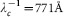, 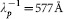, 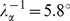, and 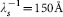. *C. elegans*: after elastic alignment; mean of three samples with ground truth by two experts; PGM parameters 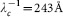, 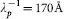, 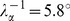, and 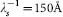.

On *C. elegans* samples, the PGM and the MWM delivered comparable results. We tested on the six *C. elegans* ground truths (3 datasets, 2 experts). The bottom part of Table 1 displays the mean of the performance measures. Both approaches performed equally well (precision 0.97, recall 0.96). We did not measure a significant difference between experts. For the PGM, all networks always converged without user input.

#### Limitations

We tested the PGM matching on the *T. brucei* sample to which the algorithm for elastic alignment had been applied. We found that it is difficult to assign the critical nodes due to the regular pattern formed by the microtubules. Figure S8 shows examples of configurations for which the algorithm requested assignments. We failed to decide whether lines were missing and which endpoints should be matched.

## Discussion

We presented computational methods for automated stitching of filaments across serial sections. Our tests on microtubule centerlines indicate good agreement of the automated results with experts' opinions for spindle samples (approximately 5% disagreeing connections per section boundary), which suggests that the proposed computational methods can be used in practice to accelerate an expert analysis. It is difficult, however, to conclude how close the results are to the true physical reality. The ground truth that we used for measuring the accuracy was created by correcting the output of the proposed computational methods. The experts might have been influenced by the output and might have decided more similar to the computational methods compared to what they would have decided if they had started from scratch. Our measurements of accuracy might, therefore, be biased in favor of our approach. On the other hand, when the computational methods disagreed with the ground truth, both choices often seemed reasonable, which suggests that some connections might be inherently difficult to decide. We also observed situations in which lines seemed to be completely missing in one section, so that no reasonable connection could be made. Since some missing lines are expected (from our experience, 4% after automatic tracing, see Weber et al. [1]), we did not correct them in our evaluation. For the microtubule arrays in *T. brucei*, the methods failed to yield satisfactory results, but experts also found it difficult to decide locally how to connect lines. We think that the proposed methods will be useful for certain experiments, like the analysis of spindle microtubules, while they might not be immediately applicable in other experiments. A good indicator whether the methods will work is probably whether an expert is able to confidently decide locally how to connect microtubule centerlines across section boundaries.

Using line orientation for the alignment has several advantages. In the experiments on robustness of the linear alignment, our algorithm for linear alignment from orientation succeeded in more cases than the other algorithms. This algorithm furthermore was the only algorithm that succeeded in computing an initial alignment for the *T. brucei* sample. Both results suggest that including orientation in the formulation stabilizes results. Orientation probably helps avoiding local extrema during optimization that would arise if only endpoint positions were used. This seems reasonable if there are preferred orientations that clearly indicated a certain alignment, such as in the *X. laevis* and *T. brucei* samples (see Figure 9). But orientation might help, because it may contain different information than position in general. The additional information might be the reason that using orientation accelerated convergence of expectation maximization for our algorithm for elastic alignment by a factor of 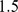 compared to the non-rigid point set registration algorithm by Myronenko and Song (Figure 4 in [28]). Finally, line orientation allows an estimation of the quality of the result by inspecting the concentration parameter 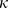 in addition to 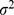. A large 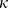 and a small 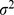 were clear indicators that the alignment succeeded. The ability to assess the result in this way without ground truth can be valuable in practice.

Using orientation for stitching has received little attention in the literature as of yet. Our approach is similar to the approach by Hogrebe et al. [55] and Dercksen et al. [23], who use segmented neuron centerlines to compute an alignment for serial sections of neurons imaged with confocal microscopy. The major difference of our approach is that it uses orientation and probabilistic methods. We think that the latter is essential here, because centerlines traced in electron tomograms are subject to noise and artifacts, and modeling uncertainty might be key to achieving reliable results.

The probabilistic approach to endpoint matching seems important. The PGM matching clearly outperformed a maximum weighted matching (MWM) if only a linear alignment was applied but no elastic alignment (Table 1, X3). Furthermore, the matching results were robust to parameter variations (Table 1, X2 and Figure 13). Both results suggest that a PGM is more reliable than a MWM and therefore the preferred approach in practice, although the performance of the MWM and the PGM matching were similar if the algorithm for elastic alignment had been applied and parameters were well chosen (Table 1, *X. laevis* X1 and *C. elegans*). For the *C. elegans* samples, the reason might be that orientation already limits the possible candidates and few ambiguities remain. The primary reason for the good performance of the PGM is probably that the pair factors enforce a coherent shift of neighboring assignments. Another reason might be that we seek user input for unclear situations. Manually assigned endpoints obviously agree with the expert's opinion in the final result. They probably also stabilize the decision on neighboring connections. Assigning endpoints manually is tedious, but in all cases, manual assignment of less than 3% of the endpoints was sufficient to achieve convergence. This seems to be an acceptable effort considering the quality of the result.

The PGM matching seems relatively robust. The maximum-likelihood estimates for the parameters were in a similar range for *X. laevis* and the *C. elegans* samples to which the algorithm for elastic alignment had been applied. The maximum-likelihood estimates for the direct and projected distances, however, were larger before elastic alignment. This is expected, because a successful alignment moves points closer and reduces the maximum-likelihood estimate, which is the mean distance between corresponding points. For similar alignment quality, similar differences in position and orientation of corresponding pairs can be expected even for different samples. The matching results were relatively unaffected by variations of the PGM parameters in our experiments on the *X. laevis* samples. Together this suggests that the same PGM parameters can probably be safely used for different samples if the alignment quality is known to be similar. But different parameters should probably be used for different experimental conditions that may introduce different errors.

The computational methods all rely on a model for facing section boundaries that makes certain assumptions about the tomograms (see details in section on computational methods): Boundaries are modeled as parallel z-planes, assuming that a skilled operator can conduct experiments such that section boundaries are relatively flat and parallel. Microtubules are modeled as straight lines, assuming that many microtubules are sufficiently perpendicular to the section boundary. Endpoints are modeled as points that are located exactly at the section boundary, assuming that more microtubules cross the section boundary than end within a section. The results suggest that the model is reasonable for the samples we used. For different conditions, however, it might be inappropriate and results should be carefully evaluated. Such conditions could be microtubules that are oriented mostly parallel to the section boundaries or many short microtubules that are likely to end within sections.

Despite the encouraging results, the limited accuracy should be considered in a subsequent analysis. Stitching errors may, in particular, bias statistics on length and number of microtubules. In our experiments, 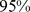 of the computed connections agreed with an expert's opinion, but 4% (*X. laevis*) and 1% (*C. elegans*) connections disagreed (Table 1). Even if disagreements counterbalanced a bit, 2% to 4% of the connections might be completely missing. This error will add up for microtubules that traverse several sections. For example, the number of microtubules might be overestimated by 80% in a stack of 20 sections in the worst case. In practice, unconnected lines should always be verified and the impact of the observed errors should be estimated for a specific analysis. Such a manual verification takes time (one hour per 500 corrections in our experience). Yet, it is still substantially less work than connecting all lines completely manually. Another benefit of a manual verification is that other errors such as line tracing errors may also be detected (from our experience 4% of the lines may be missing after automatic tracing, see Weber et al. [1]).

A potential limitation of our current approach is that the initial alignment based on the DCG might not scale for a larger number of endpoints. Although the method was successful on all *C. elegans* and *X. laevis* examples, we believe that it might be too slow for samples with more than 4000 endpoints per section. If the global structure of the sample is obvious, a simple approach for initial alignment might work, such as aligning the center and the eigenvectors of the mass distribution. If global structure is not apparent, a feature descriptor approach like Preibisch et al. [19] might be a suitable solution; or the initial alignment could be completely skipped and replaced with the algorithm for linear alignment from orientation. The algorithm could be applied from several starting points and the best solution could be automatically chosen by inspecting 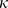 and 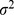.

The combination of methods that we propose might also be useful for stitching other filamentous structures. However, one major assumption of the algorithms is that lines are rather straight, which might not be the case for other applications. For curved lines, our approach could perhaps be improved by incorporating a measure of higher order line properties such as curvature. Even if lines are straight, complications might arise. For example, we believe that computing a matching for the sheets of parallel microtubules in *T. brucei* would require a different approach. Establishing correspondences is particularly challenging due to the periodicity in the sub-pellicular microtubule array.

An alignment of tomograms based on microtubules might be more accurate than an alignment based on a few manually selected landmarks. Applying the proposed methods may be useful even for samples that would otherwise be aligned manually. If tomograms contain microtubules in all relevant parts, it might make sense to segment microtubules using automatic tracing [1] and apply the proposed alignment algorithms even if microtubules are not the focus of that study.

In summary, we developed a tool to robustly stitch segmented microtubule centerlines across the gap between serial electron tomograms. We believe that our software will greatly facilitate research on microtubule organization in cell biology. Up to now, the analysis of microtubules in electron tomograms has been mostly based on observations of single sections or specimens containing only a few hundred microtubules. A quantitative analysis of length or number of microtubules was not possible for samples containing thousands of microtubules such as the spindles of *C. elegans* and *X. laevis*. The approach we introduced here should allow the analysis of microtubule centerlines over long distances across serial electron tomograms for certain structures, such as the spindle apparatus.

## Supporting Information

Figure S1 **Algorithm for linear alignment from orientation.**
(TIF)

Figure S2 **Algorithm for linear alignment from position and orientation.**
(TIF)

Figure S3
**Algorithm for elastic alignment.** The differences to the non-rigid point set registration algorithm by Myronenko and Song [28] (Figure 4 therein for two dimension 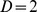) are the computation of the posterior 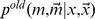 and the update of 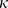.(TIF)

Figure S4
**Deformations caused by electron beam exposure.** *X. laevis* sample; scale bars 10
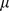
m. Left: Low-resolution image before acquisition. Middle: Same sample after an exposure time of 11 hours, which is necessary for multiple area acquisition. The sample is substantially damaged. Right: Surface that represents boundary of relevant image signal in the reconstructed volume. The surface was manually outlined using IMOD [13]. The tomogram for the surface in the topmost panel was acquired first and consequently has the least amount of deformation. The tomogram for the surface in the bottommost panel was acquired last and consequently has the largest amount of deformation.(TIF)

Figure S5 **Graphical user interface for manual inspection and correction.** The main interface elements are a perspective view of the line geometry (right) and a view that shows an oblique image slice together with surrounding lines (center). Lines from two different sections are indicated in green and blue. Black lines indicate connections across the section boundary. Endpoints are displayed in grey. The user interface elements on the left control line display parameters and support navigation, such as adjusting the views to a specific endpoint. Top: The perspective view is adjusted to show the full thickness of both sections. A line and its continuation in the neighboring section are highlighted in red. The line ends in the middle of the blue section, which probably indicates a natural microtubule end. Bottom: Closeup view as it is typically used for inspection. The user interface automatically adjusts the view to an endpoint and the surrounding lines. The operator can then inspect and make modifications. The same line as in the top panel is highlighted in red, but here only in one section.
(TIF)

Figure S6
***C. elegans*** **tomogram and ground truth for evaluation.** Top left: Slice through tomogram (scale bar 2 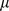
m). Bottom left: View from top on ground truth microtubule centerlines for two consecutive sections. Right: View from the right side (as indicated by the gray arrow) at the ground truth microtubule centerlines with increasingly closer views from top to bottom. The depth of view has been restricted by a clipping plane to reduce overdrawing. Connections across the section boundary are indicated in red (endpoints and connecting lines). Blue endpoints are unconnected; microtubules probably naturally end there within a section.(TIF)

Figure S7
***X. laevis*** **tomogram and ground truth for evaluation.** Top: Slice through a tomogram (scale bar 2 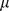
m). The orange box indicates the region for which a ground truth was prepared. Middle: View from side (as indicated by the gray arrow) at the ground truth microtubule centerlines for three consecutive sections inside the orange box. Bottom: Two closeup views. The depth of view has been restricted by a vertical slice through the tomogram to avoid overdrawing (visible in light gray in the background; some lines are partially hidden). Connections across section boundaries are indicated in red (endpoints and connecting lines). Blue endpoints are unconnected; microtubules probably naturally end there within a section, or the corresponding microtubule centerline in the next section is missing.(TIF)

Figure S8 **Alignment and matching of** ***T. brucei*** **samples.** Colors indicated different sections. Top: View from top onto results after applying the algorithm for elastic alignment (Figure S3). Some endpoints are obviously not properly aligned (yellow endpoints on left; cyan on right). The reason is probably that endpoints do not outline the same shape in each section, because the outline varies substantially from section to section. Bottom: View from side at sections for which user input during matching was requested. Red boxes indicate two critical nodes that an expert would have to assign manually. The correct continuation between sections is hard to impossible to decide locally. Bottom left: The continuation is obviously unclear. Bottom right: Assuming the small blue line that is visible close to the right image border is assigned to the yellow endpoint right above, then the assignment at the red box in the center is unclear.
(TIF)

Figure S9 **Alignment failure on sections with partial overlap.** Left: example of two sections with partial overlap. Middle: Result of applying the rigid point set registration algorithm by Myronenko and Song (Figure 2 in [28]). Right: Result of applying our algorithm for linear alignment from position and orientation (Figure S2). Both algorithms failed to compute a reasonable alignment.
(TIF)

Figure S10 **Comparison with ground truth for** ***X. laevis*** **sample.** Views at the ground truth in comparison to the PGM matching that was computed after the algorithm for elastic alignment had been applied. In-section centerlines are grey. Endpoints that agree between the ground truth and the PGM matching are indicated in blue. Endpoints that are connected in the ground truth and connected differently in the PGM matching are indicated in red. Ground truth connections are indicated in blue. Connections that are in the PGM matching but not in the ground truth are indicated in red. A red connection with two blue endpoints indicates that the connection is in the PGM matching but not in the ground truth (false positive). The depth of view has been restricted by a vertical slice through the tomogram to avoid overdrawing (visible in light gray in the background; some lines are partially hidden). A, B: all connections agree. C, D: the PGM chose different connections. E, F, G: the PGM made additional connections. H: rare situation with a line running nearly horizontally that has a long connection in the ground truth but a short connection in the PGM matching. I, J: the top and bottom section contain more lines than the middle section; lines are probably missing in the middle section. K, L: more complex situation with bundles of many parallel lines.
(TIF)

Figure S11 **Comparison with ground truth for** ***C. elegans*** **sample.** Views at the ground truth in comparison to the PGM matching that was computed after the algorithm for elastic alignment had been applied. See Figure S10 for general explanation. In addition, here a blue connection with two red endpoints indicates that the connection is in the ground truth but not in the PGM matching (false negative). A, B, D, E: the PGM chose additional connections. C: the PGM chose a different connection; rare in *C. elegans*. F, G, H: the PGM decided against connections that are in the ground truth.
(TIF)

Figure S12
**Parameter estimation for PGM factors.** Plots show normalized histograms of mutual angle 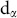 (top left), projected distance 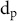 (top right), shift difference 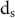 (bottom left), and direct distance 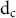 (bottom right) as obtained by analyzing connections that were verified by an expert. Blue vertical lines: Estimated means 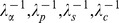. Red curve: corresponding exponential distributions 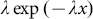. Green vertical line: Placeholder parameters 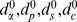 computed with a placeholder significance of 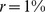. Dashed lines (bottom left) indicate distribution for our choice of 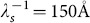 and corresponding 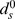.(TIF)

Figure S13
**Objective function** 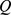 **plotted against** 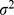 **.** Left: the objective function 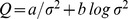 plotted against 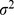 for different values of 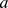 and 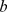. The smaller the ratio 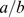, the sharper the minimum becomes. Right: the derivative 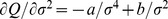 plotted against 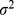 for different values of 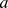 and 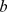. If 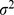 is initialized on the right side of the peak, the minimum might not be found when optimizing numerically.(TIF)

Figure S14

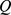
 **derivatives for minimization in algorithm for linear alignment from position and orientation (Figure S2).**
(TIF)

Text S1 **Detailed description of algorithms for fine alignment.**
(PDF)
