# Supplementary figures and images for: Automated Stitching of Microtubule Centerlines across Serial Electron Tomograms
Source: PLoS One. 2014 Dec 1;9(12):e113222. doi: 10.1371/journal.pone.0113222 (PMC4249889; doi:10.1371/journal.pone.0113222)

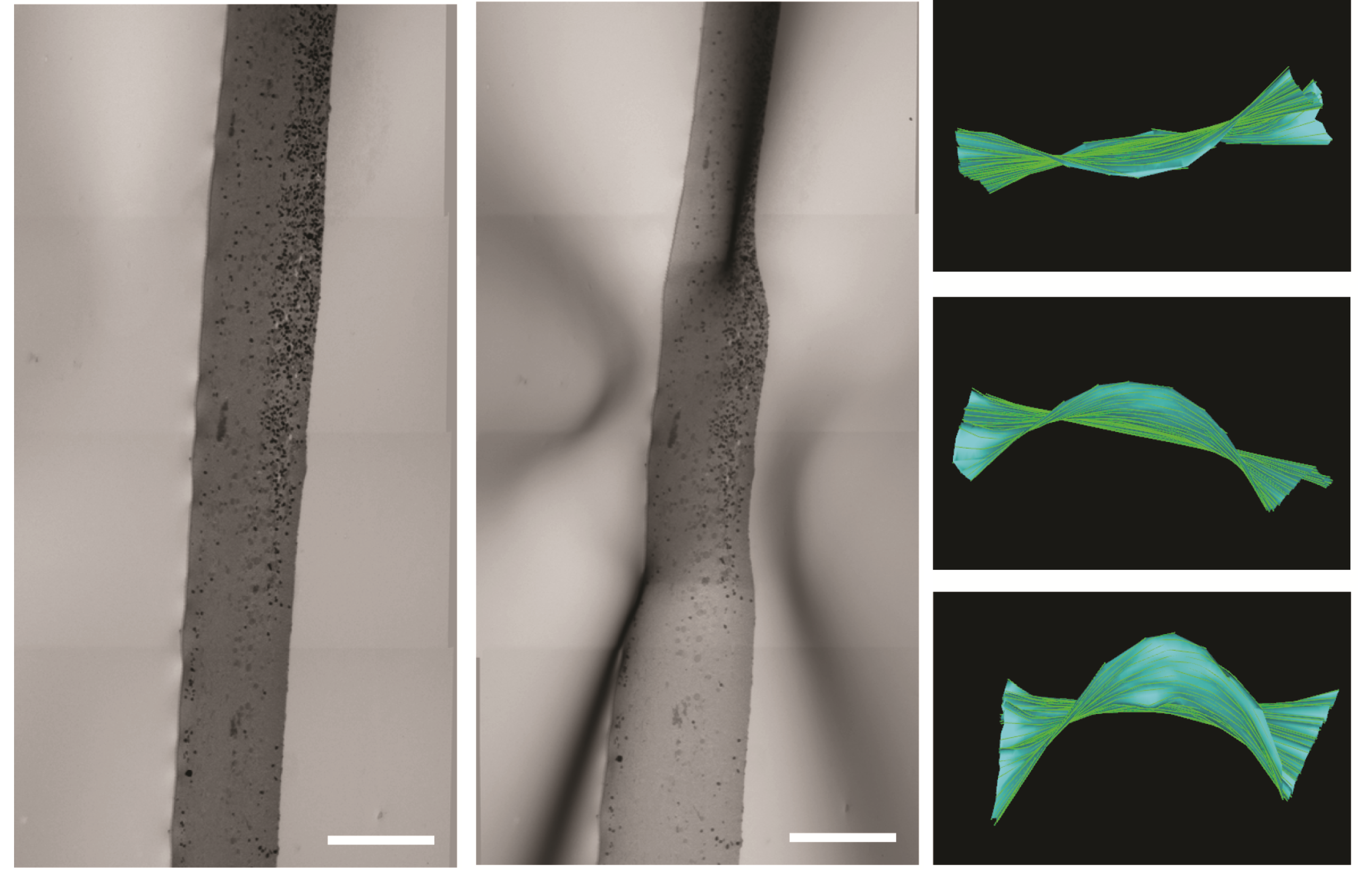

Supplement: Figure S4 — Deformations caused by electron beam exposure. X. laevis sample; scale bars 10m. Left: Low-resolution image before acquisition. Middle: Same sample after an exposure time of 11 hours, which is necessary for multiple area acquisition. The sample is substantially damaged. Right: Surface that represents boundary of relevant image signal in the reconstructed volume. The surface was manually outlined using IMOD [13]. The tomogram for the surface in the topmost panel was acquired first and consequently has the least amount of deformation. The tomogram for the surface in the bottommost panel was acquired last and consequently has the largest amount of deformation. (TIF) [file pone.0113222.s004.tif]

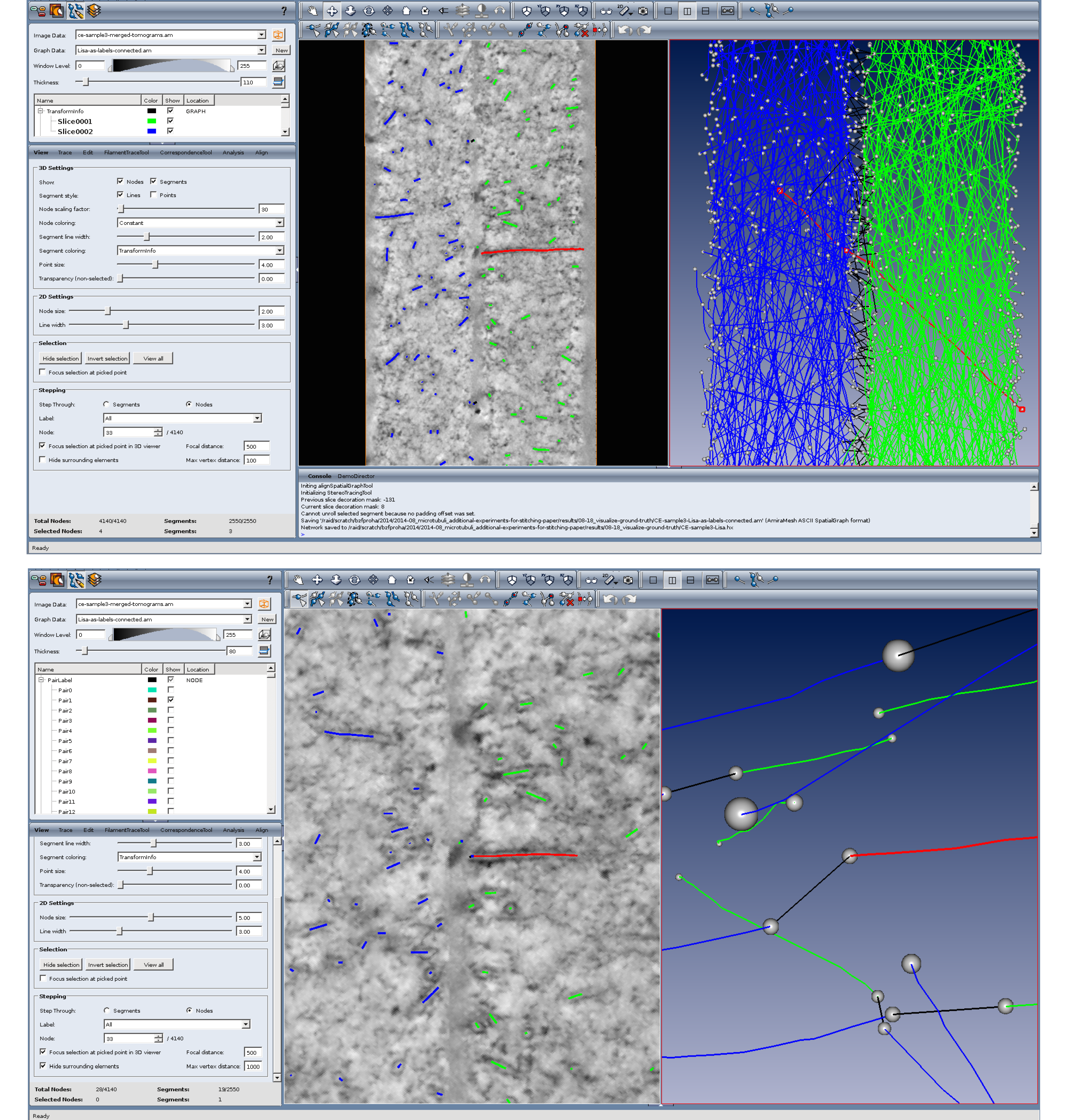

Supplement: Figure S5 — Graphical user interface for manual inspection and correction. The main interface elements are a perspective view of the line geometry (right) and a view that shows an oblique image slice together with surrounding lines (center). Lines from two different sections are indicated in green and blue. Black lines indicate connections across the section boundary. Endpoints are displayed in grey. The user interface elements on the left control line display parameters and support navigation, such as adjusting the views to a specific endpoint. Top: The perspective view is adjusted to show the full thickness of both sections. A line and its continuation in the neighboring section are highlighted in red. The line ends in the middle of the blue section, which probably indicates a natural microtubule end. Bottom: Closeup view as it is typically used for inspection. The user interface automatically adjusts the view to an endpoint and the surrounding lines. The operator can then inspect and make modifications. The same line as in the top panel is highlighted in red, but here only in one section. (TIF) [file pone.0113222.s005.tif]

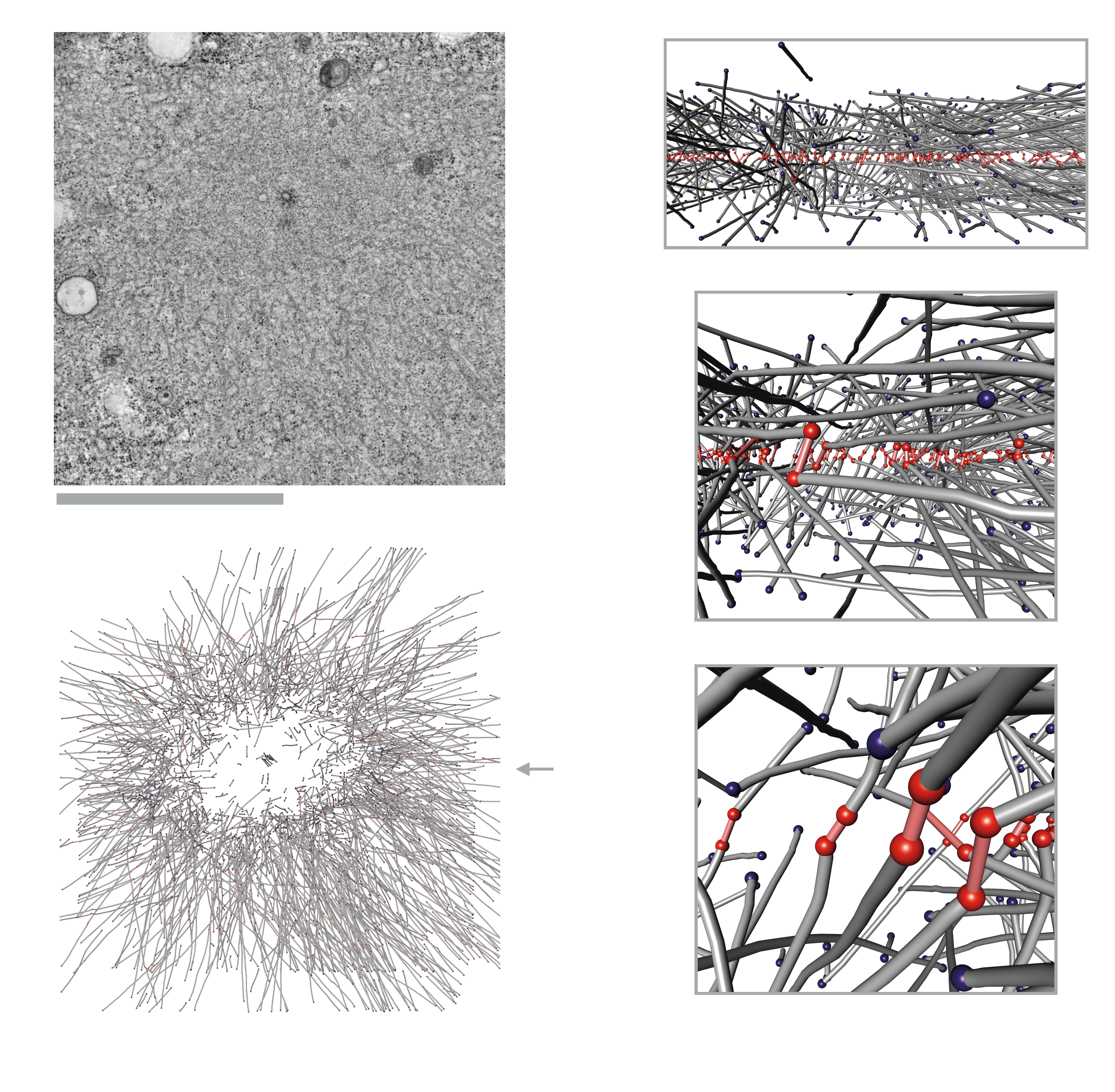

Supplement: Figure S6 — C. elegans tomogram and ground truth for evaluation. Top left: Slice through tomogram (scale bar 2 m). Bottom left: View from top on ground truth microtubule centerlines for two consecutive sections. Right: View from the right side (as indicated by the gray arrow) at the ground truth microtubule centerlines with increasingly closer views from top to bottom. The depth of view has been restricted by a clipping plane to reduce overdrawing. Connections across the section boundary are indicated in red (endpoints and connecting lines). Blue endpoints are unconnected; microtubules probably naturally end there within a section. (TIF) [file pone.0113222.s006.tif]

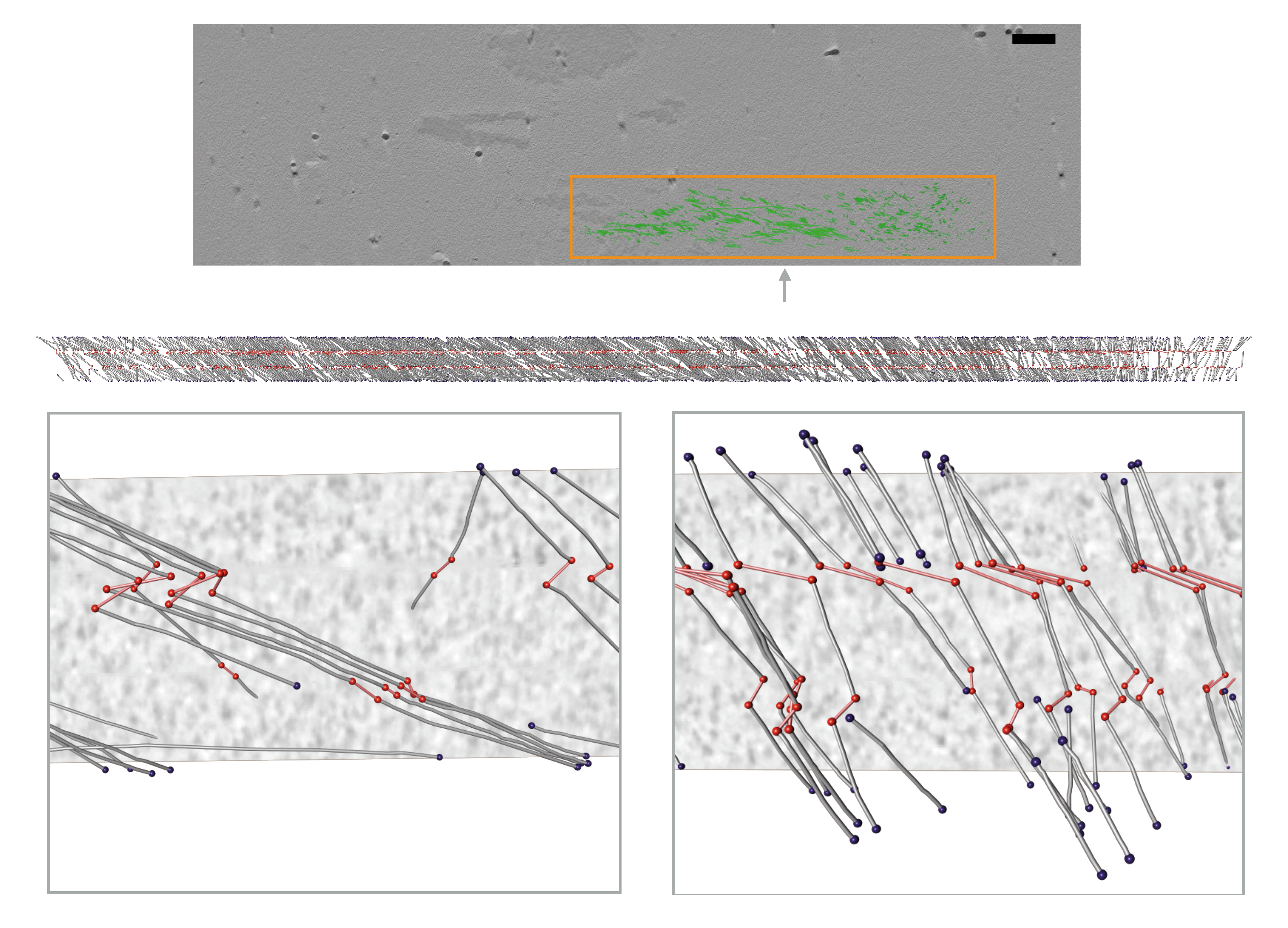

Supplement: Figure S7 — X. laevis tomogram and ground truth for evaluation. Top: Slice through a tomogram (scale bar 2 m). The orange box indicates the region for which a ground truth was prepared. Middle: View from side (as indicated by the gray arrow) at the ground truth microtubule centerlines for three consecutive sections inside the orange box. Bottom: Two closeup views. The depth of view has been restricted by a vertical slice through the tomogram to avoid overdrawing (visible in light gray in the background; some lines are partially hidden). Connections across section boundaries are indicated in red (endpoints and connecting lines). Blue endpoints are unconnected; microtubules probably naturally end there within a section, or the corresponding microtubule centerline in the next section is missing. (TIF) [file pone.0113222.s007.tif]

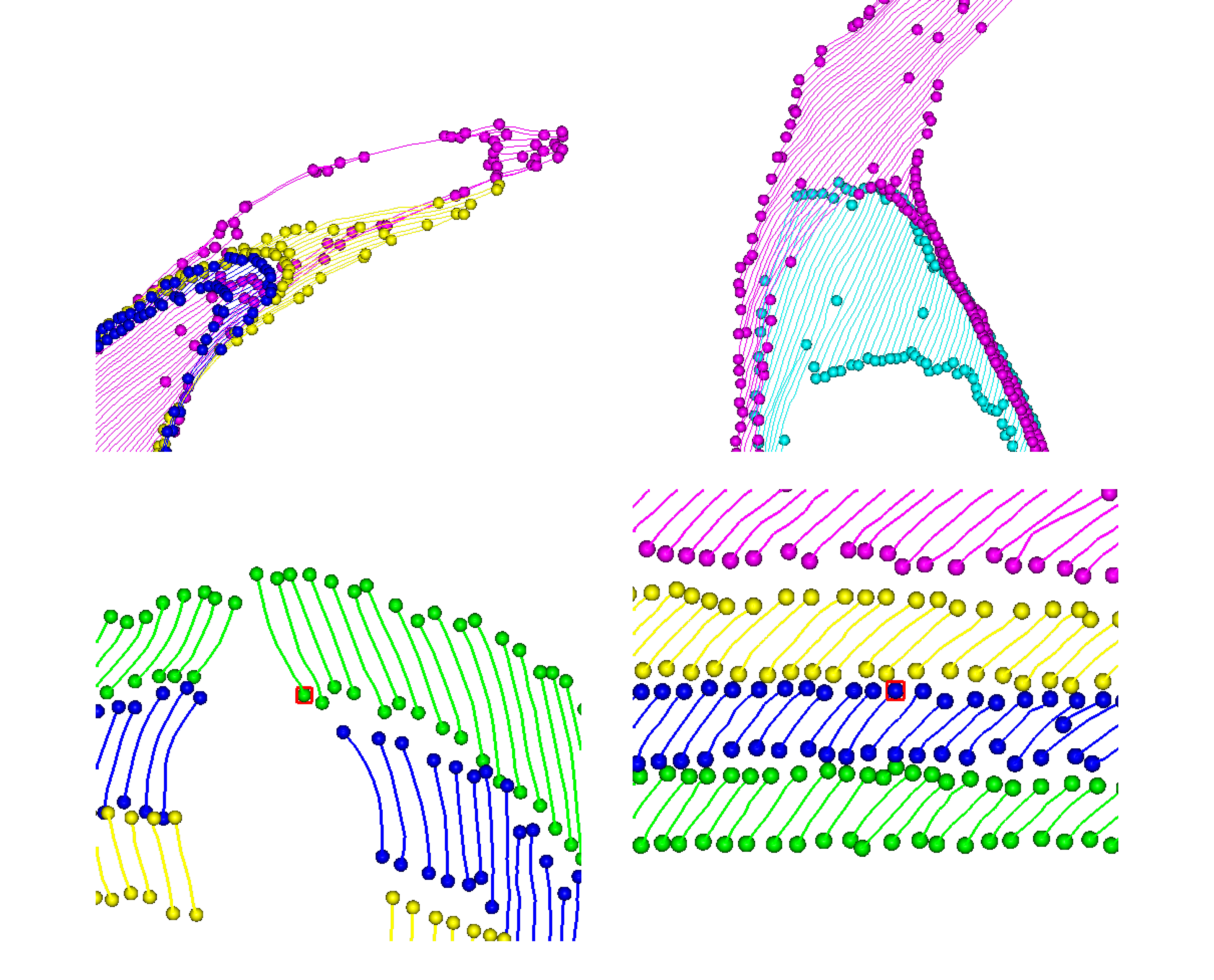

Supplement: Figure S8 — Alignment and matching of T. brucei samples. Colors indicated different sections. Top: View from top onto results after applying the algorithm for elastic alignment (Figure S3). Some endpoints are obviously not properly aligned (yellow endpoints on left; cyan on right). The reason is probably that endpoints do not outline the same shape in each section, because the outline varies substantially from section to section. Bottom: View from side at sections for which user input during matching was requested. Red boxes indicate two critical nodes that an expert would have to assign manually. The correct continuation between sections is hard to impossible to decide locally. Bottom left: The continuation is obviously unclear. Bottom right: Assuming the small blue line that is visible close to the right image border is assigned to the yellow endpoint right above, then the assignment at the red box in the center is unclear. (TIF) [file pone.0113222.s008.tif]

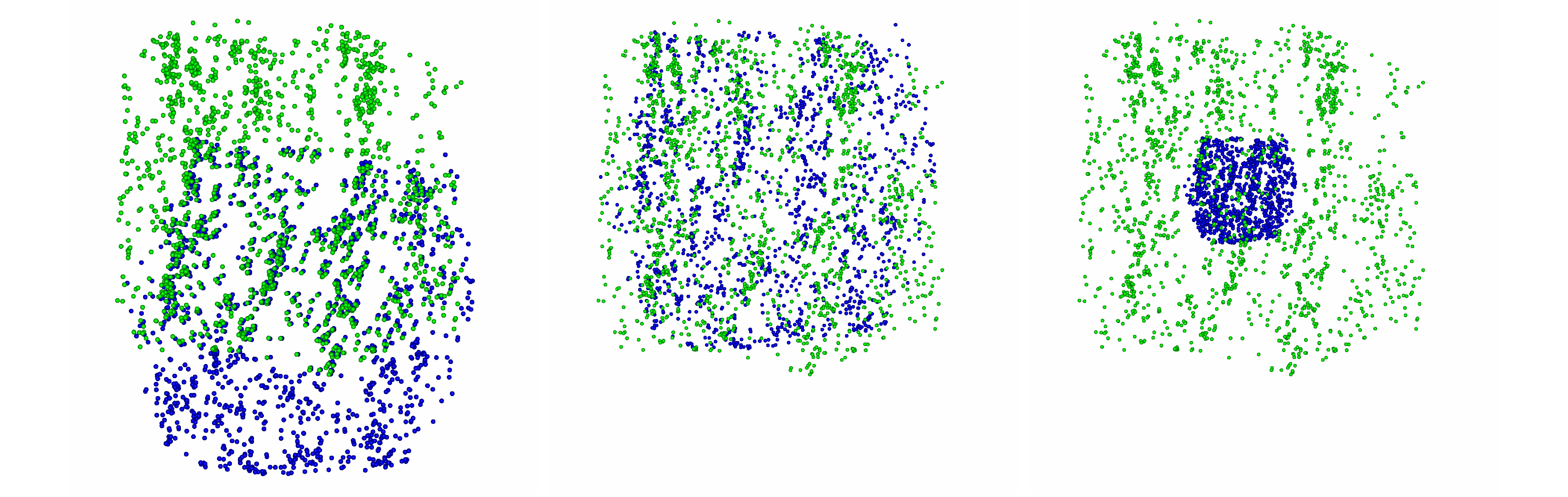

Supplement: Figure S9 — Alignment failure on sections with partial overlap. Left: example of two sections with partial overlap. Middle: Result of applying the rigid point set registration algorithm by Myronenko and Song (Figure 2 in [28]). Right: Result of applying our algorithm for linear alignment from position and orientation (Figure S2). Both algorithms failed to compute a reasonable alignment. (TIF) [file pone.0113222.s009.tif]

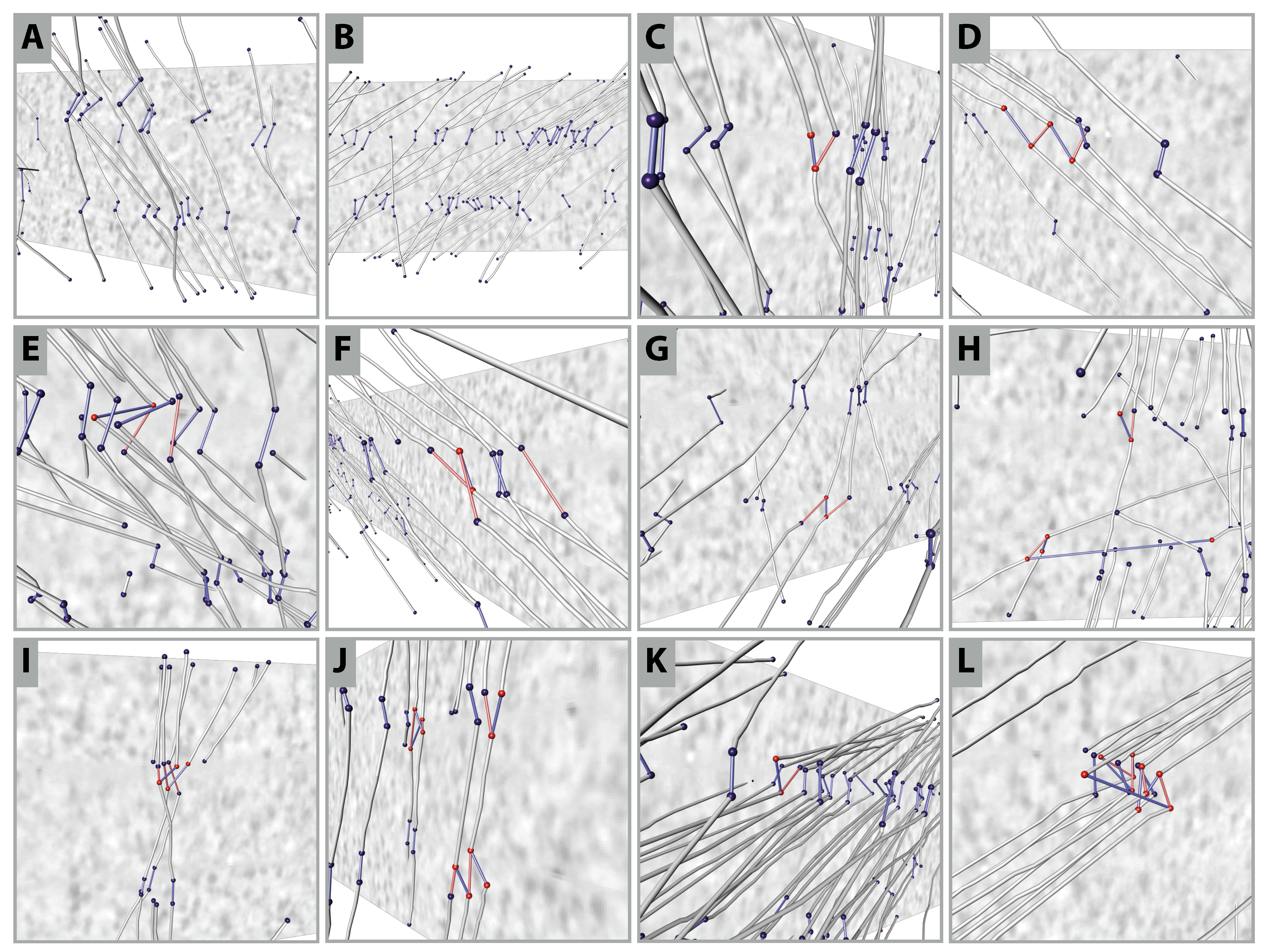

Supplement: Figure S10 — Comparison with ground truth for X. laevis sample. Views at the ground truth in comparison to the PGM matching that was computed after the algorithm for elastic alignment had been applied. In-section centerlines are grey. Endpoints that agree between the ground truth and the PGM matching are indicated in blue. Endpoints that are connected in the ground truth and connected differently in the PGM matching are indicated in red. Ground truth connections are indicated in blue. Connections that are in the PGM matching but not in the ground truth are indicated in red. A red connection with two blue endpoints indicates that the connection is in the PGM matching but not in the ground truth (false positive). The depth of view has been restricted by a vertical slice through the tomogram to avoid overdrawing (visible in light gray in the background; some lines are partially hidden). A, B: all connections agree. C, D: the PGM chose different connections. E, F, G: the PGM made additional connections. H: rare situation with a line running nearly horizontally that has a long connection in the ground truth but a short connection in the PGM matching. I, J: the top and bottom section contain more lines than the middle section; lines are probably missing in the middle section. K, L: more complex situation with bundles of many parallel lines. (TIF) [file pone.0113222.s010.tif]

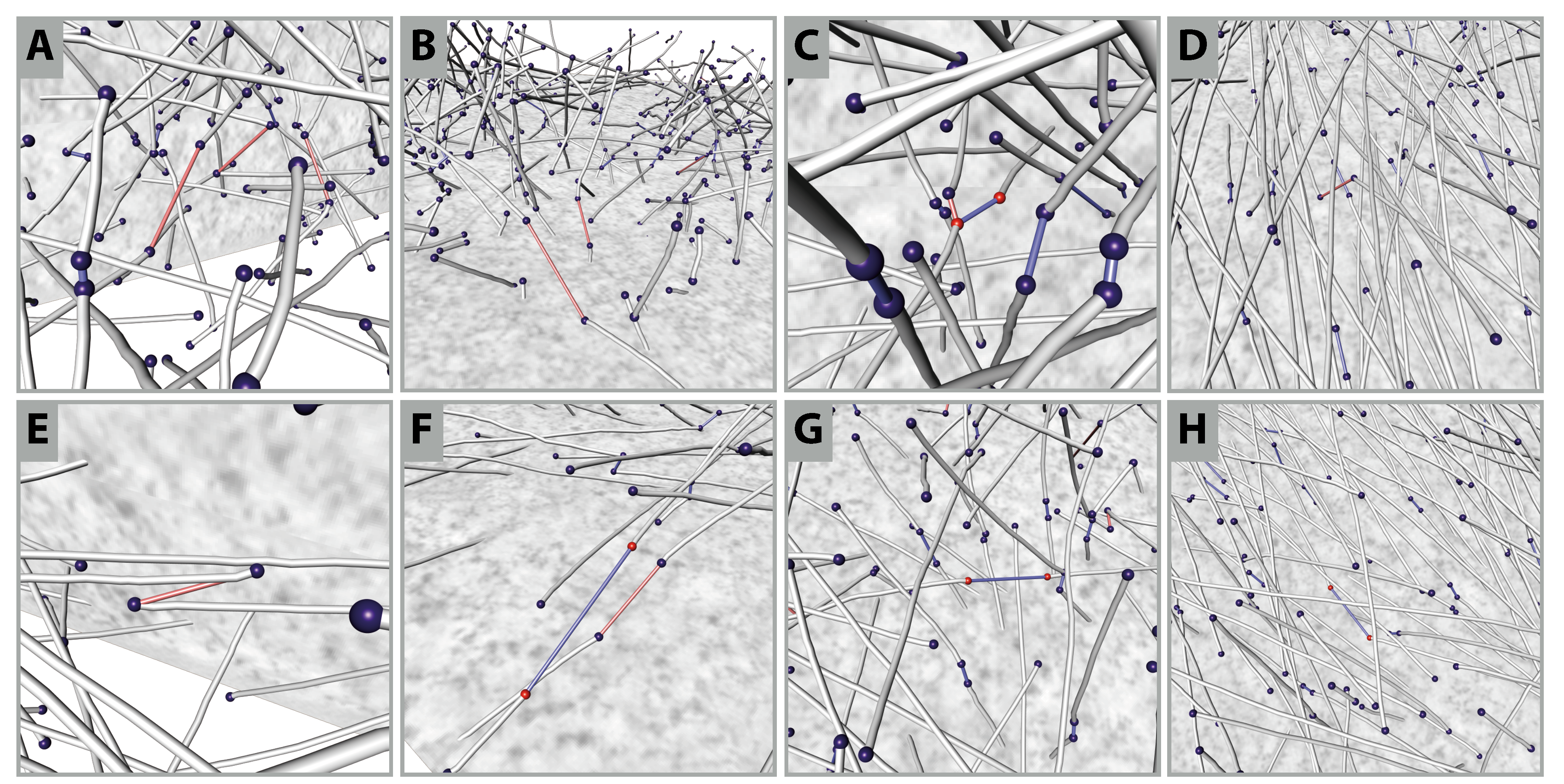

Supplement: Figure S11 — Comparison with ground truth for C. elegans sample. Views at the ground truth in comparison to the PGM matching that was computed after the algorithm for elastic alignment had been applied. See Figure S10 for general explanation. In addition, here a blue connection with two red endpoints indicates that the connection is in the ground truth but not in the PGM matching (false negative). A, B, D, E: the PGM chose additional connections. C: the PGM chose a different connection; rare in C. elegans. F, G, H: the PGM decided against connections that are in the ground truth. (TIF) [file pone.0113222.s011.tif]

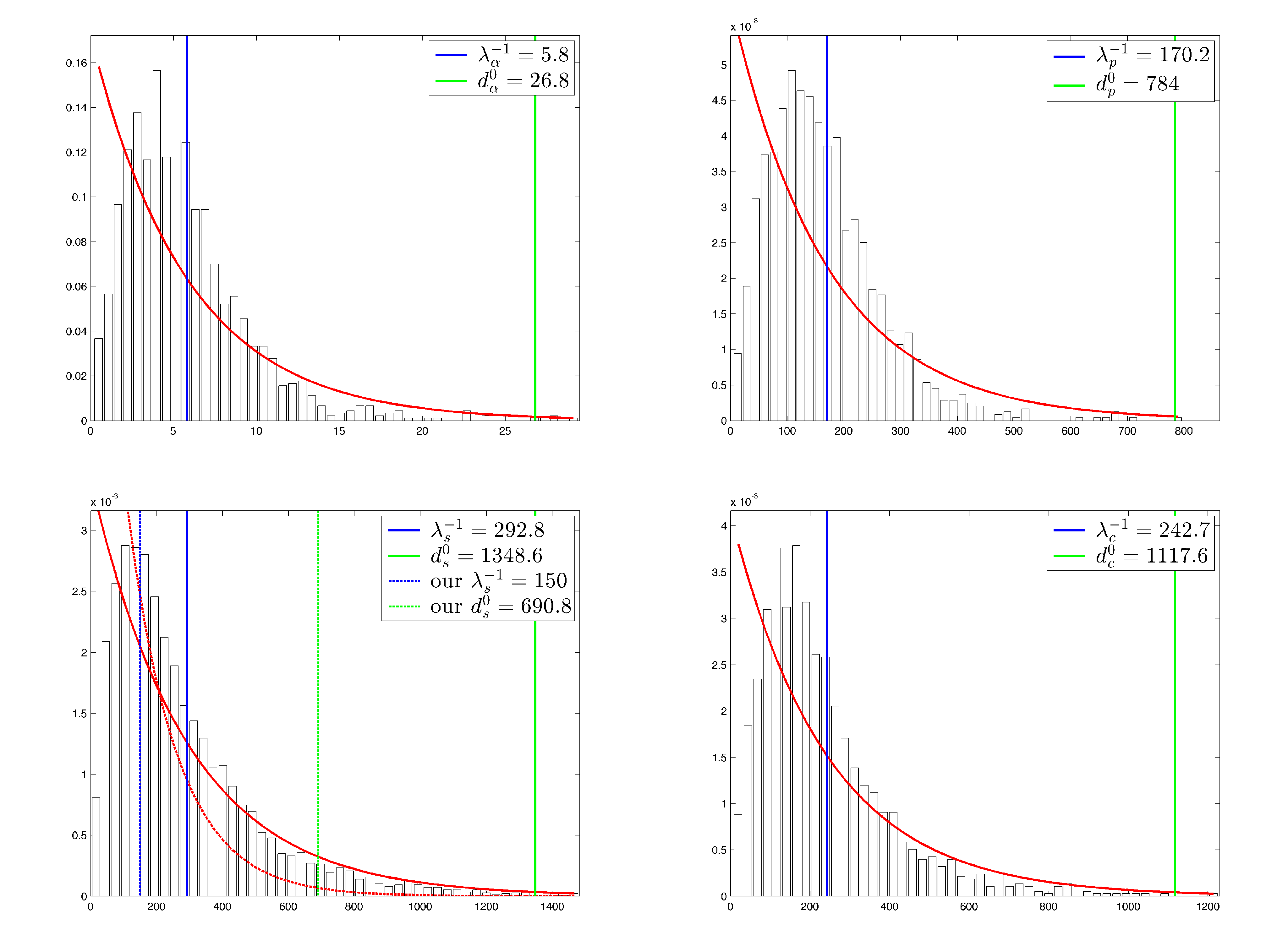

Supplement: Figure S12 — Parameter estimation for PGM factors. Plots show normalized histograms of mutual angle (top left), projected distance (top right), shift difference (bottom left), and direct distance (bottom right) as obtained by analyzing connections that were verified by an expert. Blue vertical lines: Estimated means . Red curve: corresponding exponential distributions . Green vertical line: Placeholder parameters computed with a placeholder significance of . Dashed lines (bottom left) indicate distribution for our choice of and corresponding . (TIF) [file pone.0113222.s012.tif]

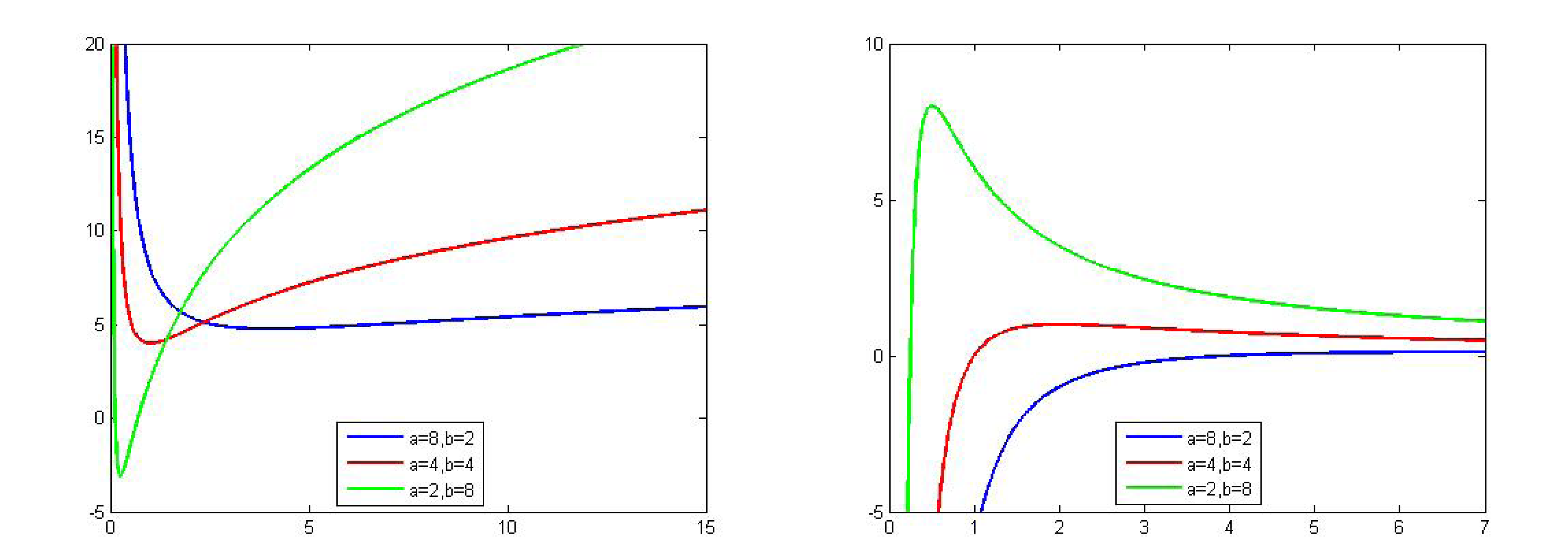

Supplement: Figure S13 — Objective function plotted against . Left: the objective function plotted against for different values of and . The smaller the ratio , the sharper the minimum becomes. Right: the derivative plotted against for different values of and . If is initialized on the right side of the peak, the minimum might not be found when optimizing numerically. (TIF) [file pone.0113222.s013.tif]
