# Supplementary material for: Automated Stitching of Microtubule Centerlines across Serial Electron Tomograms
Source: PLoS One. 2014 Dec 1;9(12):e113222. doi: 10.1371/journal.pone.0113222 (PMC4249889; doi:10.1371/journal.pone.0113222)
Supplement: Text S1 — Detailed description of algorithms for fine alignment. (PDF) [file pone.0113222.s015.pdf]

Supporting Information: Automated stitching of microtubule centerlines across serial electron tomograms

See main manuscript for references and figures.

Algorithms for fine alignment

This section describes in detail the algorithms for fine alignment. The method uses the expectation maximization algorithm. To apply expectation maximization, we derive 1) the update equations for the posterior $P(\mathbf{m}|\mathbf{x})$ in the E-step and 2) the update equations for the distribution and transformation parameters in the M-step, which is done by minimizing the expectation of the complete negative log-likelihood function L.

We give the detailed derivation of the update equations for each of the three methods and summarize the algorithms in Figures S2, S3 and S4 in the style of Myronenko and Song [28].

Notation In addition to the notation defined in the main text we will use the following notation: For two sets of endpoints $x_1, ...x_N$ and $y_1, ...y_M$ the matrix $\mathbf{X}$ contains all endpoint positions in X as rows $\mathbf{X} = (x_1, .., x_N)^\top$ and likewise $\mathbf{Y} = (y_1, .., y_M)^\top$.

$\mathbf{P}$ denotes an $M \times N$ matrix that contains the values of the posterior $P(\mathbf{m}|\mathbf{x})$ for all possible pairs x_n, y_m. Furthermore, we write $N_P = \sum_{m,n=1}^{M,N} P(\mathbf{m}|\mathbf{x})$.

The operator diag(v) creates a diagonal matrix from a vector $v \in R^D$ by $\text{diag}(v) = \begin{pmatrix} v_1 & \dots & 0 \\ \vdots & \ddots & \vdots \\ 0 & \dots & v_D \end{pmatrix}$.

We denote a vector containing only ones as $\mathbf{1}$.

Further prerequisites To account for outliers, Myronenko and Song introduce an additional term $P(\mathbf{x}, M+1) = 1/N$ to the joint distribution. This outlier term can be weighted with $0 \leq w \leq 1$. The marginal probability of $\mathbf{x}$ then takes the form:

$$P(\mathbf{x}) = \sum_{m=1}^{M+1} p(m)P(\mathbf{x}|m) = \frac{1-w}{M} \sum_{m=1}^{M} P(\mathbf{x}|m) + \frac{w}{N}. \tag{12}$$

We use the same outlier term in all three settings.

For convenience, we will often write sums as matrices using the following equalities:

$$\sum_{m,n=1}^{M,N} P(m|x_n)x_n = \mathbf{X}^\top \mathbf{P}^\top \mathbf{1} \tag{13}$$

$$\sum_{m,n=1}^{M,N} P(m|x_n)y_m = \mathbf{Y}^\top \mathbf{P} \mathbf{1} \tag{14}$$

$$\sum_{m,n=1}^{M,N} P(m|x_n)x_n^\top x_n = \text{tr}(\mathbf{X}^\top \,\text{diag}(\mathbf{P}^\top \mathbf{1})\mathbf{X}) \tag{15}$$

$$\sum_{m,n=1}^{M,N} P(m|x_n)x_n x_n^\top = \mathbf{X}^\top \,\text{diag}(\mathbf{P}^\top \mathbf{1})\mathbf{X} \tag{16}$$

$$\sum_{m,n=1}^{M,N} P(m|x_n) y_m^\top y_m = \mathrm{tr}(\mathbf{Y}^\top \operatorname{diag}(\mathbf{P1})\mathbf{Y}) \tag{17}$$

$$\sum_{m,n=1}^{M,N} P(m|x_n) y_m y_m^\top = \mathbf{Y}^\top \operatorname{diag}(\mathbf{P1})\mathbf{Y} \tag{18}$$

$$\sum_{m,n=1}^{M,N} P(m|x_n) x_n^\top R y_m = \mathrm{tr}(\mathbf{X}^\top \mathbf{P}^\top \mathbf{Y} R^\top) \tag{19}$$

$$\sum_{m,n=1}^{M,N} P(m|x_n) x_n y_m^\top R^\top = \mathbf{X}^\top \mathbf{P}^\top \mathbf{Y} R^\top \tag{20}$$

Algorithm for linear alignment from orientation

E-step The posterior $P(\vec{\mathbf{m}}|\vec{\mathbf{x}})$ is computed by

$$P(\vec{\mathbf{m}}|\vec{\mathbf{x}}) = \frac{P(\vec{\mathbf{m}}, \vec{\mathbf{x}})}{P(\vec{\mathbf{x}})}. \tag{21}$$

To compute $P(\vec{\mathbf{m}}, \vec{\mathbf{x}})$, we add an outlier term $P(\vec{\mathbf{x}}|M+1) = 1/N$ and weight the priors with $0 \le w \le 1$ so that $P(\mathbf{m}) = (1-w)/M$ and $P(M+1) = w/N$. $P(\vec{\mathbf{m}}, \vec{\mathbf{x}})$ can be computed from the priors and $P(\vec{\mathbf{x}}|\vec{\mathbf{m}})$ which is given by Equation 6 for $m \le M$. The joint distribution becomes:

$$P(\vec{\mathbf{m}}, \vec{\mathbf{x}}) = P(\vec{\mathbf{m}})P(\vec{\mathbf{x}}|\vec{\mathbf{m}}) = \frac{(1-w)}{M} \frac{\kappa}{2\pi(e^\kappa - e^{-\kappa})} \exp(\kappa (R\vec{y}_m)^\top \vec{\mathbf{x}}), \text{ for all } m \le M \text{ and} \tag{22}$$

$$P(\vec{\mathbf{x}}, M+1) = w/N, \text{ for } m = M+1. \tag{23}$$

$P(\vec{\mathbf{x}})$ can be computed from $P(\vec{\mathbf{m}}, \vec{\mathbf{x}})$ by marginalizing out $\vec{\mathbf{m}}$:

$$P(\vec{\mathbf{x}}) = P(\vec{\mathbf{x}}, M+1) + \sum_{m=1}^{M} P(\vec{\mathbf{m}}, \vec{\mathbf{x}}) = \frac{w}{N} + (1-w)\sum_{k=1}^{M} \frac{\kappa}{2M\pi(e^\kappa - e^{-\kappa})} \exp(\kappa (R\vec{y}_k)^\top \vec{\mathbf{x}}) \tag{24}$$

The posterior becomes

$$P(\vec{\mathbf{m}}|\vec{\mathbf{x}}) = \frac{P(\vec{\mathbf{m}}, \vec{\mathbf{x}})}{P(\vec{\mathbf{x}})} = \frac{\exp(\kappa \vec{x}_n^\top R \vec{y}_m)}{\sum_{k=1}^{M} \exp(\kappa \vec{x}_n^\top R \vec{y}_k) + \frac{wM}{(1-w)N} \frac{2\pi(\exp(\kappa) - \exp(-\kappa))}{\kappa}} \tag{25}$$

M-step In the M-step, we must write out the expectation of the negative log-likelihood function Q (Equation 3) and minimize it with respect to the transformation parameters R and the distribution parameter κ. Q becomes:

$$\begin{aligned} Q(R, \kappa) = -\kappa \sum_{m,n=1}^{M,N} p^{old}(m|\vec{x}_n) \vec{x}_n^\top R \vec{y}_m - N_P \log(\frac{\kappa}{e^\kappa - e^{-\kappa}}) + \text{const.} = \\ -\kappa \,\mathrm{tr}((\vec{\mathbf{X}}^\top \mathbf{P}^\top \vec{\mathbf{Y}})^\top R) - N_P \log(\frac{\kappa}{e^\kappa - e^{-\kappa}}) + \text{const.} \end{aligned} \tag{26}$$

We split the term $\mathrm{tr}((\vec{\mathbf{X}}^\top \mathbf{P}^\top \vec{\mathbf{Y}})^\top R)$ into terms that are dependent on and independent of $\tilde{R}$:

$$\mathrm{tr}((\vec{\mathbf{X}}^\top \mathbf{P}^\top \vec{\mathbf{Y}})^\top R) = \mathrm{tr}((\vec{\mathbf{X}}_{2d}^\top \mathbf{P}^\top \vec{\mathbf{Y}}_{2d})^\top \tilde{R}) + \vec{\mathbf{X}}_z^\top \mathbf{P}^\top \vec{\mathbf{Y}}_z \tag{27}$$

where $\vec{\mathbf{X}}_{2d}$ is a matrix containing the first two columns of $\vec{\mathbf{X}}$ and $\vec{\mathbf{X}}_z$ contains the last column of $\vec{\mathbf{X}}$.

The optimal rotation can be obtained with a Singular Value Decomposition from the term $\vec{\mathbf{X}}_{2d}^\top \mathbf{P}^\top \vec{\mathbf{Y}}_{2d}$ (equivalently to Myronenko and Song [28], see Umeyama [42] and Myronenko and Song [43] for details on the method). κ cannot be obtained in closed form, but it can be computed with Newton's method. We iterate

$$\kappa = \kappa^{old} - \frac{\partial Q}{\partial \kappa} / \frac{\partial^2 Q}{\partial^2 \kappa} \text{ until convergence, where}$$

$$\frac{\partial Q}{\partial \kappa} = -\operatorname{tr}((\vec{\mathbf{X}}_{2d}^\top \mathbf{P}^\top \vec{\mathbf{Y}}_{2d})^\top \tilde{R}) - \operatorname{tr}(\vec{\mathbf{X}}_z^\top \mathbf{P}^\top \vec{\mathbf{Y}}_z) - N_P(\frac{1}{\kappa} - \coth \kappa), \text{ and} \tag{28}$$

$$\frac{\partial^2 Q}{\partial^2 \kappa} = -N_P(-\frac{1}{\kappa^2} + \frac{1}{\sinh \kappa^2}). \tag{29}$$

Because we only compute a rotation, the endpoints are not aligned. To align them, we first note that even though we did not explicitly take the positions into account, the likelihood that two orientations match also applies to the corresponding endpoint positions. To compute an alignment of the positions, we compute the squared distances of the positions, which we weight by the posterior computed from the obtained optimal parameters Θ^*, $P(\vec{\mathbf{m}}|\vec{\mathbf{x}}, \Theta^*)$:

$$S = \sum_{m,n=1}^{M,N} p(\vec{y}_m|\vec{x}_n, \Theta^*)|x_n - (\tilde{R}y_m + t)|^2.$$

To minimize the distances, we first eliminate the brackets and take the derivative with respect to t:

$$S = \sum_{m,n=1}^{M,N} p(\vec{y}_m|\vec{x}_n, \Theta^*)(-2x_n + 2\tilde{R}y_m + 2t).$$

Solving for t yields:

$$t = \frac{1}{N_P} \sum_{m,n=1}^{M,N} p(\vec{y}_m|\vec{x}_n, \Theta^*)x_n - \frac{1}{N_P} \sum_{m,n=1}^{M,N} p(\vec{y}_m|\vec{x}_n, \Theta^*)\tilde{R}y_m = \mu_x - \tilde{R}\mu_y.$$

We summarize the algorithm in Figure S2.

Algorithm for linear alignment from position and orientation

E-step The joint distribution of $\mathbf{x}, \vec{\mathbf{x}}$ and $\mathbf{m}, \vec{\mathbf{m}}$ is (Equation 2 and 6):

$$P(\mathbf{x}, \vec{\mathbf{x}}, \mathbf{m}, \vec{\mathbf{m}}) = \mathcal{N}(\mathbf{x}; \mathbf{m}, \sigma^2)\mathcal{F}_3(\vec{\mathbf{x}}; \vec{\mathbf{m}}, \kappa)\frac{1}{M}\delta_{\text{ind}(\mathbf{m}),\text{ind}(\vec{\mathbf{m}})} \tag{30}$$

To compute the posterior $P(\mathbf{m}, \vec{\mathbf{m}}|\mathbf{x}, \vec{\mathbf{x}})$, we must again apply Bayes' rule. We add an outlier term as in the previous algorithm and weight the priors as before with $0 \leq w \leq 1$. The posterior then is:

$$\begin{aligned} P(\mathbf{m}, \vec{\mathbf{m}}|\mathbf{x}, \vec{\mathbf{x}}) &= \frac{P(\mathbf{x}, \vec{\mathbf{x}}, \mathbf{m}, \vec{\mathbf{m}})}{P(\mathbf{x}, \vec{\mathbf{x}})} = \\ &\frac{(1-w)\mathcal{N}(\mathbf{x}; \mathbf{m}, \sigma^2)\mathcal{F}(\vec{\mathbf{x}}; \vec{\mathbf{m}}, \kappa)\frac{1}{M}\delta_{\text{ind}(\mathbf{m}),\text{ind}(\vec{\mathbf{m}})}}{\frac{w}{N} + \sum_{k=1}^{M} \mathcal{N}(\mathbf{x}; y_k, \sigma^2)\mathcal{F}(\vec{\mathbf{x}}; \vec{y}_k, \kappa)\frac{1}{M}\delta_{k,k}} = \\ &\frac{\exp(-\frac{1}{2\sigma^2}||x_n - s\tilde{R}y_m - t||^2)\exp(\kappa \vec{x}_n^\top R\vec{y}_m)}{\sum_{k=1}^{M} \exp(-\frac{1}{2\sigma^2}||x_n - s\tilde{R}y_k - t||^2)\exp(\kappa \vec{x}_n^\top R\vec{y}_k)) + \frac{wM}{(1-w)N}\frac{4\pi^2\sigma^2(\exp(\kappa)-\exp(-\kappa))}{\kappa}} \end{aligned} \tag{31}$$

M-step The expectation of the complete negative log-likelihood function is given by

$$
\begin{aligned}
Q(s,\tilde{R},t,\sigma^2,\kappa) =& \frac{1}{2\sigma^2}\sum_{m,n=1}^{M,N} p^{old}(y,\vec{y}|x,\vec{x})||x_n - s\tilde{R}y_m - t||^2 + N_P \log\sigma \\
& -\kappa \sum_{m,n=1}^{M,N} p^{old}(y,\vec{y}|x,\vec{x})\vec{x}_n^\top R\vec{y}_m - N_P \log(\frac{\kappa}{e^\kappa - e^{-\kappa}}) + \text{const.}
\end{aligned}
\tag{32}
$$

To solve for each of the parameters during the M-Step, we first compute t by taking the partial derivative of Q with respect to t, as we did in the last section, to obtain (equivalently to Myronenko and Song [28])

$$
t = \frac{1}{N_P}\sum_{m,n=1}^{M,N} p^{old}(y_m,\vec{y}_m|x_n,\vec{x}_n)x_n - \frac{1}{N_P}\sum_{m,n=1}^{M,N} p^{old}(y_m,\vec{y}_m|x_n,\vec{x}_n)\tilde{R}y_n = \mu_x - s\tilde{R}\mu_y \tag{33}
$$

We then plug t back into Equation 32. The log-likelihood becomes

$$
\begin{aligned}
Q(s,\tilde{R},\sigma^2,\kappa) =& -\frac{1}{2\sigma^2}\sum_{m,n=1}^{M,N} p^{old}(y,\vec{y}|x,\vec{x})||\hat{x}_n - s\tilde{R}\hat{y}_m||^2 + \frac{N_P}{2}\log\sigma \\
& -\kappa \sum_{m,n=1}^{M,N} p^{old}(y,\vec{y}|x,\vec{x})\vec{x}_n^\top R\vec{y}_m - N_P \log(\frac{\kappa}{e^\kappa - e^{-\kappa}}) + \text{const.},
\end{aligned}
\tag{34}
$$

where $\hat{x}_n = x_n - \mu_x$ and $\hat{y}_m = y_m - \mu_y$. We then write out $||\hat{x}_n - s\tilde{R}\hat{y}_m||^2$ as a product $(\hat{x}_n - s\tilde{R}\hat{y}_m)^\top(\hat{x}_n - s\tilde{R}\hat{y}_m)$, eliminate the brackets and write Q in matrix form:

$$
\begin{aligned}
Q(s,\tilde{R},\sigma^2,\kappa) = +\frac{1}{2\sigma^2}(&\text{tr}(\hat{\mathbf{X}}^\top \text{diag}(\mathbf{P}^\top\mathbf{1})\hat{\mathbf{X}}) - 2s\cdot\text{tr}((\hat{\mathbf{X}}^\top\mathbf{P}^\top\hat{\mathbf{Y}})^\top\tilde{R}) + s^2\,\text{tr}(\hat{\mathbf{Y}}^\top \text{diag}(\mathbf{P1})\hat{\mathbf{Y}})) - \\
& -\kappa\,\text{tr}((\vec{\mathbf{X}}^\top\mathbf{P}^\top\vec{\mathbf{Y}})^\top R) + N_P\log\sigma^2 - N_P\log(\frac{\kappa}{e^\kappa - e^{-\kappa}}) + \text{const.}
\end{aligned}
\tag{35}
$$

We could not find a closed form solution for any of the parameters. Instead we search the optimum for the parameters numerically, except for t, which we already eliminated. In general, using numerical optimization in this case is problematic due to the constraints $\tilde{R}^\top\tilde{R} = \mathbf{I}$ and $\det(\tilde{R}) = 1$. In our case, however, the rotation matrix is only two-dimensional and can therefore be parameterized by a single angle ρ that can be treated as one additional parameter. To find a numerical solution for this problem, usually first and second derivatives of the objective function are required. These are not hard to find from Equation 35, but it is tedious work. For the convenience of the reader, we give details on the derivative with respect to ρ below (Section Q derivatives) and list all first and second order derivatives in Figure S15. Figure S3 summarizes the algorithm.

The numerical optimization in the M-step is an unfortunate consequence of the scaling parameter s. Without the scaling, the objective function Q could probably be minimized by formulating the transformation as dual quaternions and solving the transformation parameters as described by Walker [44]. While this still would not provide a final optimization in closed form, it might be possible to calculate closed form update equations for a generalized expectation maximization. Note that we cannot use other approaches that use scaled vectors such as Wells [26], because our orientations are unit vectors.

Algorithm for elastic alignment

For the elastic transformation model, y is transformed via a displacement vector $\nu(y)$ (Myronenko and Song [28])

$$
T(Y,\nu) = Y + \nu(Y). \tag{36}
$$

Here, the task is to find a function ν that gives enough flexibility to capture the deformation of the data but still maintains the overall neighborhood structure of the points.

Myronenko and Song derive the displacement vectors by solving the regularized expectation of the negative log-likelihood function

$$f(\nu, \sigma^2) = Q_c(\nu, \sigma^2) + \frac{\lambda}{2}\Phi(\nu), \tag{37}$$

where Φ penalizes high frequencies of ν. See [28] for a detailed derivation of the update equations for the M-step. The resulting vectors $\nu(y)$ are the product of an $M \times M$ smoothing matrix $\mathbf{G}$ and, for the 2D case, an $M \times 2$ matrix $\mathbf{W}$ by $\mathcal{T}(y_m, \Theta) = y_m + (\mathbf{G}(m, \cdot)\mathbf{W})^\top$, where $\mathbf{G}(m, \cdot)$ denotes the mth row of $\mathbf{G}$. The transformation parameters Θ here are the entries in the matrix $\mathbf{W}$, whereas $\mathbf{G}$ is initialized once and stays fixed.

M-step Coupling the transformation of positions and orientations is not obvious, because the transformation is formulated as position displacement vectors ν (Equation 36) and the rotational part of the transformation is not explicitly accessible. In practice, we assume that the elastic transformation contains only a minimal rotation that can be ignored, because we have applied a linear alignment before. With this assumption we can rewrite Equation 37 with the orientation as

$$f(\nu, \sigma^2, \kappa) = Q_c(\nu, \sigma^2) + \frac{\lambda}{2}\Phi(\nu) + Q_d(\kappa), \text{ with} \tag{38}$$

$$Q_d(\kappa) = -\kappa \sum_{m,n=1}^{M,N} p^{old}(y_m, \vec{y}_m | x_n, \vec{x}_n)\vec{x}_n^\top \vec{y}_m - N_P \log(\frac{\kappa}{e^\kappa - e^{-\kappa}}) + \text{const., and} \tag{39}$$

$$Q_c(\sigma^2) = \frac{1}{2\sigma^2} \sum_{m,n=1}^{M,N} p^{old}(y_m, \vec{y}_m | x_n, \vec{x}_n)||x_n - (y_m - \nu(y_m))||^2 + N_P \log \sigma^2 + \text{const.} \tag{40}$$

Because Q_d is independent of the displacement vectors μ, the optimization can be performed exactly as described in Myronenko and Song [28]. The only difference here is that the prior $P(\mathbf{m}, \vec{\mathbf{m}}|\mathbf{x}, \vec{\mathbf{x}})$ depends on κ, which must also be updated at each M-step. κ can be updated in each iteration again with Newton's method. With this strategy, the orientation only influences the result via the posterior $P^{old}(\mathbf{m}, \vec{\mathbf{m}}|\mathbf{x}, \vec{\mathbf{x}})$.

E-step The posterior again is computed by Bayes' rule

$$\begin{aligned} P(\mathbf{m}, \vec{\mathbf{m}}|\mathbf{x}, \vec{\mathbf{x}}) = \frac{P(\mathbf{x}, \vec{\mathbf{x}}, \mathbf{m}, \vec{\mathbf{m}})}{P(\mathbf{x}, \vec{\mathbf{x}})} = \\ \frac{\exp(-\frac{1}{2\sigma^2}||x - (y_m + (\mathbf{G}(m, \cdot)\mathbf{W})^\top)||^2)\exp(\kappa \vec{x}^\top \vec{y}_m)}{\sum_{k=1}^{M} \exp(-\frac{1}{2\sigma^2}||x - (y_k + (\mathbf{G}(m, \cdot)\mathbf{W})^\top)||^2)\exp(\kappa \vec{x}^\top \vec{y}_k)) + \frac{wM}{(1-w)N}\frac{4\pi^2\sigma^2(\exp(\kappa) - \exp(-\kappa))}{\kappa}} \end{aligned} \tag{41}$$

The algorithm is summarized in Figure S4.

Implementation details

To optimize Q for the linear alignment from position and orientation (Algorithm S3), we use IPOpt [48], a free optimization library. As the starting point for the optimization, we use the parameters from the previous step, except for σ^2. In our experiments, σ^2 is not optimized correctly if the initial value is placed on the wrong side of the peak of the derivative of Q (see Figure S14). To avoid this, we initialize σ^2 as

$$\sigma^2 = \frac{1}{2N_P}(\text{tr}(\hat{\mathbf{X}}^\top \operatorname{diag}(\mathbf{P}^\top \mathbf{1})\hat{\mathbf{X}}) - 2s \operatorname{tr}((\hat{\mathbf{X}}^\top \mathbf{P}^\top \hat{\mathbf{Y}})^\top \tilde{R}) + s^2 \operatorname{tr}(\hat{\mathbf{Y}}^\top \operatorname{diag}(\mathbf{P1})\hat{\mathbf{Y}})),$$

which would be the minimum of σ^2 if the other parameters were fixed. In our experiments this was sufficient to guarantee that the initial σ^2 is placed on the right side.

Furthermore, the smaller σ becomes the sharper the peak that is the minimum of the objective function becomes (see Figure S14). This sharp peak might make it hard to estimate a reasonable step size when optimization is done numerically. We believe that this is the reason why IPOpt does not always find an optimum in later expectation maximization iterations in our implementation. However, for our test data, we found that about 4000 iterations in IPOpt were sufficient to find a new parameter set that decreased the log-likelihood.

The running time of the algorithm for elastic alignment is $O(M^3)$. It can can reduced to linear by computing P with a fast Gauss transform and G by a low rank approximation as described by Myronenko and Song [28]. We did not implement this technique, which is why our implementation has cubic running time.

Q derivatives

Splitting the negative log-likelihood function (Equation 35) again into terms dependent and independent of $\tilde{R}$ (by Equation 4), Q can be written as :

$$\begin{aligned} Q(s,\tilde{R},\sigma^2,\kappa) = \frac{1}{2\sigma^2}(&\operatorname{tr}(\hat{\mathbf{X}}^\top \operatorname{diag}(\mathbf{P}^\top\mathbf{1})\hat{\mathbf{X}}) - 2s\cdot\operatorname{tr}((\hat{\mathbf{X}}^\top\mathbf{P}^\top\hat{\mathbf{Y}})^\top\tilde{R}) + s^2\operatorname{tr}(\hat{\mathbf{Y}}^\top\operatorname{diag}(\mathbf{P1})\hat{\mathbf{Y}}))- \\ &\kappa(\operatorname{tr}((\vec{\mathbf{X}}_{2d}{}^\top\mathbf{P}^\top\vec{\mathbf{Y}}_{2d})^\top\tilde{R}) + \vec{\mathbf{X}}_z{}^\top\mathbf{P}^\top\vec{\mathbf{Y}}_z) \\ &N_P\log\sigma^2 - N_P\log(\frac{\kappa}{e^\kappa - e^{-\kappa}}) \end{aligned} \tag{42}$$

To find the optimal parameters $\tilde{R},\sigma^2,\kappa$, we must find the partial derivatives of Q. To simplify notation, we define $A = \hat{\mathbf{X}}^\top\operatorname{diag}(\mathbf{P}^\top\mathbf{1})\hat{\mathbf{X}}$, $B = \hat{\mathbf{X}}^\top\mathbf{P}^\top\hat{\mathbf{Y}}$, $C = \hat{\mathbf{Y}}^\top\operatorname{diag}(\mathbf{P1})\hat{\mathbf{Y}}$, $D = \vec{\mathbf{X}}_{2d}{}^\top\mathbf{P}^\top\vec{\mathbf{Y}}_{2d}$ and $E = \vec{\mathbf{X}}_z{}^\top\mathbf{P}^\top\vec{\mathbf{Y}}_z$, and write again Q as

$$\begin{aligned} Q(s,\tilde{R},\sigma^2,\kappa) = \frac{1}{2\sigma^2}(&\operatorname{tr}(A) - 2s\cdot\operatorname{tr}(B^\top\tilde{R}) + s^2\operatorname{tr}(C))- \\ &\kappa(\operatorname{tr}(D^\top\tilde{R}) + \operatorname{tr}(E))+ \\ &N_P\log\sigma^2 - N_P\log(\frac{\kappa}{2\pi(e^\kappa - e^{-\kappa})}) + \text{const.} \end{aligned} \tag{43}$$

To compute the partial derivative with respect to $\tilde{R}$, we note that $\tilde{R}$ is parametrized via one rotation angle ρ as $\tilde{R} = \begin{pmatrix}\cos\rho & -\sin\rho \\ \sin\rho & \cos\rho\end{pmatrix}$ in the 2d case. Writing the trace as a sum and taking partial derivatives with respect to this angle we find that

$$\frac{\partial\operatorname{tr}(K^\top\tilde{R})}{\partial\rho} = \operatorname{tr}(K^\top\frac{\partial\tilde{R}}{\partial\rho}) \tag{44}$$

and

$$\frac{\partial\tilde{R}}{\partial\rho} = \tilde{R}' = \begin{pmatrix}-\sin\rho & -\cos\rho \\ \cos\rho & -\sin\rho\end{pmatrix} \tag{45}$$

The second derivative can be found equivalently.

$$\frac{\partial^2\tilde{R}}{\partial^2\rho^2} = \tilde{R}'' = \begin{pmatrix}-\cos\rho & \sin\rho \\ -\sin\rho & -\cos\rho\end{pmatrix} \tag{46}$$

We give the partial derivatives of Equation 43 with respect to s,ρ,σ^2 and κ in Figure S15.
